# Effect of *Thuja occidentalis* L. Essential Oil Combined with Diatomite Against Selected Pests

**DOI:** 10.3390/molecules30153300

**Published:** 2025-08-06

**Authors:** Janina Gospodarek, Elżbieta Boligłowa, Krzysztof Gondek, Krzysztof Smoroń, Iwona B. Paśmionka

**Affiliations:** 1 Department of Microbiology and Biomonitoring, University of Agriculture, al. A. Mickiewicza 21, 31-120 Krakow, Poland; elzbieta.boliglowa@urk.edu.pl (E.B.); iwona.pasmionka@urk.edu.pl (I.B.P.); 2 Department of Agricultural and Environmental Chemistry, University of Agriculture, al. A. Mickiewicza 21, 31-120 Krakow, Poland; krzysztof.gondek@urk.edu.pl; 3 Specialized Mining Company “Górtech” Sp. z o.o., ul. Galicyjska 1/43b, 31-586 Krakow, Poland; k.smoron@diato.pl

**Keywords:** black bean aphid, Colorado potato beetle, pea leaf weevil, eastern white cedar, natural products, field crop pests, foraging inhibition

## Abstract

Combining products of natural origin with different mechanisms of action on insect herbivores may provide an alternative among methods of plant protection against pests that are less risky for the environment. The aim of the study was to evaluate the effectiveness of mixtures of *Thuja occidentalis* L. essential oil and diatomite (EO + DE) compared to each substance separately in reducing economically important pests such as black bean aphid (BBA) *Aphis fabae* Scop., Colorado potato beetle (CPB) *Leptinotarsa decemlineata* Say., and pea leaf weevil (PLW) *Sitona lineatus* L. The effects on mortality (all pests) and foraging intensity (CPB and PLW) were tested. The improvement in effectiveness using a mixture of EO + DE versus single components against BBA was dose- and the developmental stage-dependent. The effect of enhancing CPB foraging inhibition through DE addition was obtained at a concentration of 0.2% EO (both females and males of CPB) and 0.5% EO (males) in no-choice experiments. In choice experiments, mixtures EO + DE with both 0.2% and 0.5% EO concentrations resulted in a significant reduction in CPB foraging. A significant strengthening effect of EO 0.5% through the addition of DE at a dose of 10% against PLW males was observed in the no-choice experiment, while, when the beetles had a choice, the synergistic effect of a mixture of EO 0.5% and DE 10% was also apparent in females. In conclusion, the use of DE mixtures with EO from *T. occidentalis* appears to be a promising strategy. The results support the idea of not using doses of EO higher than 0.5%.

## 1. Introduction

The urge to replace chemical methods of plant protection against pests with methods that are less risky for the environment, including humans, generates the need to look for new solutions in this area. One such solution may be to combine products of natural origin with different mechanisms of action on insect herbivores.

Essential oils (EOs) are known for a number of uses, including as insecticides [1,2]. Since EOs are mixtures of many components, the development of herbivore resistance to the active substances they contain may be inhibited. Their greater environmental safety is also emphasized [1,2,3]. The downside of using EOs under field conditions is the rapid volatilization of active ingredients and susceptibility to degradation, which significantly reduces the persistence of their effects [4].

Diatomite (DE) is one of the materials with high environmental potential [5,6]. It is formed from the siliceous shells of monocellular algae, of which the main constituents are silica hydrates with varying degrees of hydration (SiO_2__nH_2_O) [7]. Among the mineral constituents, quartz, feldspars, glauconite, and calcite are present, in addition to amorphous silica [8,9]. In terms of chemical composition, DE contains the following: 55–95% SiO_2_; 5.5–14.4% Al_2_O_3_; 0.5–10% Fe_2_O_3_; 0.2–4.0% MgO and CaO [5]. The frequently reported characteristics of DE include low density, porosity, and good wettability [5]. DE is indicated as an effective material for the treatment of water, wastewater, or the immobilization of heavy metals in soil [8,10]. DE significantly increases the stability of soil aggregates and the moisture and field capacity in sandy soil [11]. It has the potential to improve soil microbial activity as well [12]. Recently, the importance of DE in agriculture has also been increasing, including as a substance with insecticidal properties [13]. Desiccation and abrasion are mentioned as the main mechanisms of DE action against pests [13,14]. The advantages of using DE are that it is a natural substance with low environmental toxicity, that it can be easily separated from the product to which it is applied (e.g., grains) or from plants (by rain), and that it is also readily available [13]. Furthermore, it is believed that, due to its mechanism of action, it will not cause pests to become rapidly resistant [13]. As such, it has been extensively studied to date for use against storage pests [15,16] and arthropods of urban interest [17]. The downside of DE is the observed decrease in efficacy under higher moisture conditions, which is a serious limitation for use in field operations. DE in a mixture with water is not as effective as in a dust formulation [18]. In spite of promising laboratory results for the application of DE into the soil against crop pests, use under field conditions against, e.g., aphids in wheat crops has not yielded satisfactory results [19].

In view of the aforementioned, it is reasonable to look for mixtures that would combine the insecticidal/foraging inhibition efficacy of its individual components and, at the same time, reduce the limitations of each of these substances used separately—i.e., the rapid volatilization and decomposition of EOs under open-air conditions and the reduced efficacy of DE at higher humidity. The combination of EOs with DE, due to the strong sorption properties of the latter, can prolong the effect of EOs in the open field and, at the same time, the EOs present in the mixture will sustain the effect of DE in higher humidity regimes. The addition of DE to EO can also reduce the potential phytotoxic effect of EO. Such combinations have already been studied for the control and deterrence of storage pests [20]. Binary mixtures of DE with the chitin synthesis inhibitors chlorfluazuron and hexaflumuron caused higher mortality of *Callosobruchus maculatus* (F.) and *C. chinensis* (L.) than individual treatments [15]. *Sitophilus zeamais* (Motschulsky) showed an average mortality after 24 h exposure to the mixture of DE and *Ammoides verticillata* EO (1 mg of DE/8 µL EO/cm^2^) of 80%, while the use of DE alone caused a mortality of 40%, and the LC_50_ for EO alone was 1.99 mg/cm^3^ [20]. A mixture of DE with powders of *Eugenia aromatica* and *Moringa oleifera* controlled adults of *Sitophilus granarius* (L.), *Tribolium castaneum* (Herbst), and *Acanthoscelides obtectus* (Say) faster than botanical powders alone [21]. The addition of monoterpenoids (cinnamaldehyde or eugenol) increased the toxicity of DE against *Sitophilus oryzae* (L.) compared to DE alone, but no such synergistic effect was observed for another storage pest *Callosobruchus maculatus* (Fabr.) [22]. The authors, therefore, emphasize that the effectiveness of DE can be increased by adding the specific substances against a particular pest. Regarding the use of DE mixtures with plant-based substances under field conditions, trials have so far been undertaken against pests in orchards (*Drosophila suzukii* Matsumura) and vineyards (*Planococcus ficus* Signoret) [23,24]. However, there are limited data in the available literature regarding pests of agricultural crops.

EO from *Thuja occidentalis* L. has so far been investigated for use in reducing storage pests [25,26,27,28,29], sanitary insects [30], orchard pests [31], and agricultural plant pests [32], with significant efficacy highlighted. The main components of this EO are α-thujone (20.1–61.0%), beyerene (1.1–12.8%), β-thujone (3.6–10.7%), sabinene (3.0–9.3%), and fenchone (4.9–7.7%). Depending on the origin of the plant, it may also contain significant amounts of constituents such as myrcene, bornyl acetate, and terpinyl acetate [32,33,34,35,36].

To evaluate the potential of DE and EO from *T. occidentalis* mixtures, three insect species of high importance as crop pests were selected: the black bean aphid (BBA) (*Aphis fabae* Scopoli, 1763), the pea leaf weevil (PLW) (*Sitona lineatus* Linnaeus, 1758), and the Colorado potato beetle (CPB) (*Leptinotarsa decemlineata* Say, 1824). BBA is a polyphagous pest that feeds on many economically important crops such as beetroot, broad bean, and maize, causing plant death and transmitting dangerous viruses [37,38]. PLW in its imaginal form eats the edges of the leaf blade of legumes (particularly dangerous to emerging plants), while, in its larval stage, it damages the root nodules of these plants [39,40]. CPB, on the other hand, is a major pest of potato, causing multi-million-dollar losses worldwide each year [41].

The aim of the study was to evaluate the effectiveness of mixtures of *T. occidentalis* essential oil and diatomite (EO + DE) compared to each substance separately in reducing the survival of BBA nymphs and wingless females, as well as the food consumption of PLW and CPB adults. Due to the higher sensitivity of aphids to EOs and DE reported in the literature than that of beetles [13,42], two doses of DE (5% and 10%) and three concentrations of EO (0.2%, 0.5% and 1.0%) were tested for BBA, while only the higher dose of DE (10%) and three concentrations of EO (0.2%, 0.5% and 1.0%) were tested for PLW and CPB. The use of higher doses of EO against PLW and CPB was abandoned due to the possible phytotoxic effect of EO at doses higher than 1% (effect observed in preliminary experiments). For PLW and CPB, the possibility of a deterrent effect of lower doses of EO (0.2% and 0.5%) and the same doses of EO mixed with DE in 10% concentration, as well as of DE 10% alone in choice experiments, was also tested.

We assumed the following hypotheses:

1. EO + DE will cause higher herbivore mortality than its components alone.
2. EO + DE will exhibit a stronger and more persistent foraging inhibition effect against PLW and CPB.
3. The effect will be dose- and development stage-/sex- dependent.

## 2. Results

### 2.1. Aphis fabae Scop.

A statistical analysis (two-way ANOVA) showed a significant effect of both EO and DE on the survival of wingless females of BBA (results of multivariate tests: for EO F = 22.51, *p* < 0.001; for DE F = 2.89, *p* < 0.001; for EO × DE F = 1.86, *p* < 0.001; results for individual observation terms in Table S1). In addition, a significant interaction between the two factors was noted. For this reason, data are presented to show these possible interactions (factors: EO × DE). The use of DE alone generally did not result in a significant increase in mortality of BBA females for most of the time (compared to the control, i.e., EO 0 DE 0 treatment) (Table 1). A significant insecticidal effect was only observed after 66, 78, and 90 h with DE at both concentrations and after 54 and 102 h for the DE 10 and DE 5 treatments, respectively. However, the increase in mortality of BBA females did not exceed 24%. The addition of DE at a concentration of 5% to EO from *T. occidentalis* at a concentration of 0.2% resulted in the increased survival of wingless females; however, the effect was only significant at the final stage of the experiment (after 90 and 102 h). The use of a higher dose of DE—10%—mitigated this antagonistic effect of DE (i.e., there were no significant differences between the EO 0.2 DE 0 and EO 0.2 DE 10 treatments). It should be emphasized, however, that both EO alone at a concentration of 0.2% and in combination with DE 10 from 54 h onwards resulted in significantly lower aphid survival compared to the control. For the EO 0.2 DE 5 treatment, a similar effect was recorded after 66, 90, 78, and 114 h of the experiment. In the case of the higher EO dose—0.5%, the DE addition enhanced the effect of EO from 54 h of the experiment onwards, with the higher DE dose (10%) causing higher female mortality. For the highest concentration of EO—1%, which killed aphids 100% immediately after application, the DE admixture showed some protective effect against aphids (antagonistic effect of DE), but only during the initial period of the experiment—i.e., up to 42 h. However, the percentage of females that survived the application of EO 1 DE 5 and EO 1 DE 10 was 3–5 times lower at this time compared to the control.

**Figure 1:**
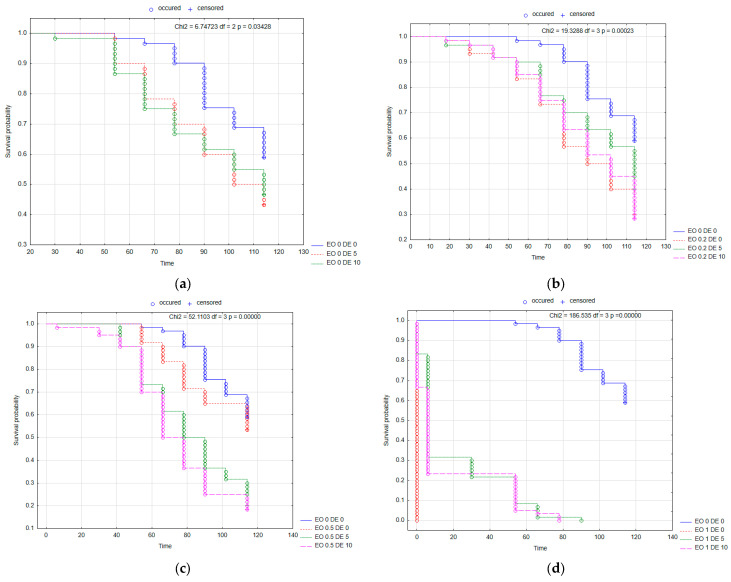
Kaplan–Meier survival curves of *Aphis fabae* Scop. wingless females after the application of EO from *T. occidentalis* L. and diatomite: effect of DE alone (**a**); effect of EO in concentration 0.2% with and without DE (**b**); effect of EO in concentration 0.5% with and without DE (**c**); effect of EO in concentration 1% with and without DE (**d**). For treatment descriptions, see Table 1.

**Figure 2:**
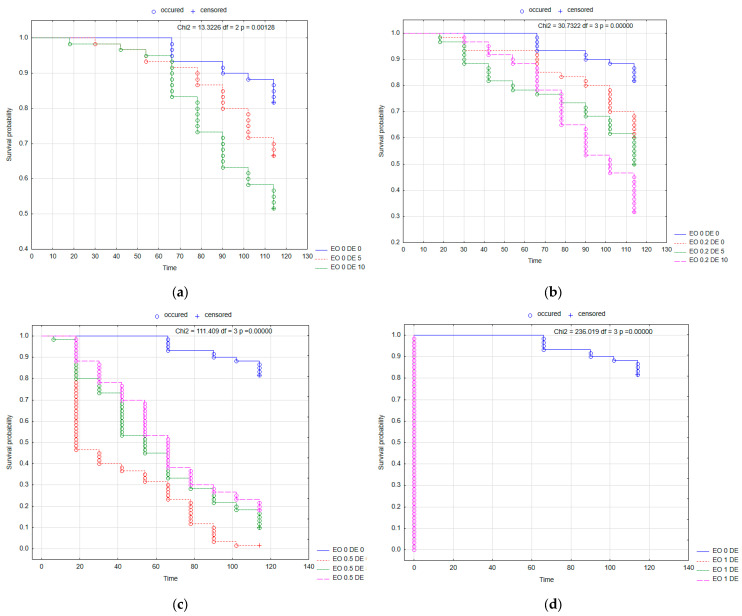
Kaplan–Meier survival curves of *Aphis fabae* Scop. nymphs after the application of EO from *T. occidentalis* L. and diatomite: effect of DE alone (**a**); effect of EO in concentration 0.2% with and without DE (**b**); effect of EO in concentration 0.5% with and without DE (**c**); effect of EO in concentration 1% with and without DE (**d**). For treatment descriptions, see Table 1.

The above-described relationships are confirmed by the survival probability curves of BBE females in the individual treatments calculated using the Kaplan–Meier method (Figure 1). In each of the treatments analyzed, the probability of survival was lower than in the control (EO 0 DE 0). An antagonistic effect of the addition of DE in EO 0.2 and EO 1 treatments (Figure 2b,d) could be observed, as well as a synergistic effect in EO 0.5 treatment (Figure 2c).

In the case of BBA nymphs, two-way ANOVA also showed a significant effect of EO and DE, as well as an interaction of both factors (multivariate test results: for EO F = 136.51, *p* < 0.001; for DE F = 3.10, *p* < 0.001; for EO × DE F = 2.37, *p* < 0.001, Table S2). However, the response of BBA nymphs was slightly different from that of females (Table 2). DE alone caused a significant increase in nymph mortality only after 78 h of the experiment (effect significant until 114 h) for a concentration of 10% (by about 21–31%) and after 114 h for a concentration of 5% (by about 25%) compared to the control. The addition of DE at a concentration of 10% to 0.2% *T. occidentalis* EO increased the mortality of BBA nymphs starting from 78 h of the experiment by about 23–27% compared to the treatment with 0.2% EO alone. The addition of DE at a concentration of 5% did not cause a significant difference compared to 0.2% EO alone; however, it slightly enhanced the insecticidal effect of 0.2% EO, so that, starting from 42 h of the experiment, survival in the EO 0.2 DE 5 treatment was significantly lower compared to the control. For EO at a concentration of 0.5%, the addition of DE generally resulted in reduced nymph mortality at most observation dates. The antagonistic effect of DE increased with a higher DE dose. The difference between EO 0.5 DE 0 and EO 0.5 DE 5 ranged from 7 to 34%, and between EO 0.5 DE 0 and EO 0.5 DE 10 from 15 to 42%, depending on the observation term. No antagonistic effect of DE was found at the highest concentration of EO (i.e., 1%). Regardless of whether EO was used alone or with the addition of DE, 100% nymph mortality was recorded.

The probability of survival of BBE nymphs in each treatment, as in the case of wingless females, was lower than in the control (EO 0 DE 0) (Figure 2). An antagonistic effect of the DE addition (the stronger the higher the DE dose) could be observed for EO 0.5% (Figure 2c), and a synergistic effect was recorded for EO 0.2% (Figure 2b).

Calculated LC_50_ values for *T. occidentalis* EO when used alone and with the addition of DE at concentrations of 5% and 10% (Table 3) indicate the higher efficacy of EO + DE mixtures compared to EO alone at later times after application. Initially, LC_50_ values for wingless females were higher when EO + DE mixtures were used compared to EO alone, but from 54 h of the experiment onwards, this relationship was reversed. The effect was stronger for the higher dose of DE. For nymphs, the reduction in LC_50_ values occurred later than for wingless females—after 78 h for DE 10 and after 114 h for DE 5.

### 2.2. Leptinotarsa decemlineata Say.

#### 2.2.1. No-Choice Experiment

**Figure 3:**
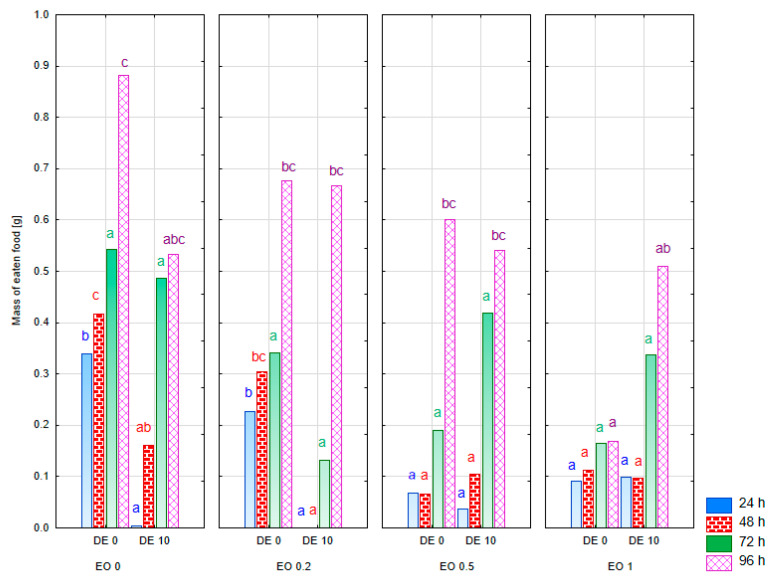
Mass of food eaten by one live female of *Leptinotarsa decemlineata* Say. [g] after the application of EO from *T. occidentalis* L. and diatomite (measurement in 24 h intervals from 24 to 96 h in relation to the initial mass of food each time; bars in the same color correspond to the respective observation dates; factors: EO × DE). For treatment descriptions, see Table 1. Different letters for values at specific time (the same color of font) mark statistically significant differences (*p* ≤ 0.05).

In the experiment where CPB was offered either the treated food or the control food separately, a significant effect of both EO and DE was found, as well as an interaction of both factors (results of multivariate tests for females: EO − F = 4.01, *p* < 0.001; DE − F = 13.91, *p* < 0.001; EO × DE − F = 4.52, *p* < 0.001 and for males: EO − F = 3.55, *p* < 0.001; DE − F = 50.62, *p* < 0.001; EO × DE − F = 3.85, *p* < 0.001). The results of the statistical analysis for the individual observation times in the case of CPB females showed no effect of the studied factors after 72 h, while, at the other times, either a significant effect of DE (after 24 and 48 h) or EO (after 48 and 96 h) or the interaction of both factors (after 24 and 48 h) was recorded (Table S3). No significant antagonistic effect of DE was noted in any of the cases studied. DE alone at a concentration of 10% resulted in a reduction in the mass of food eaten compared to the control only during the initial 48 h (Figure 3). The addition of DE at a concentration of 10% to 0.2% EO from *T. occidentalis* caused a significant inhibition of foraging by CPB females, persisting for the initial 48 h of the experiment and making the effect significant relative to the control, while EO 0.2% alone did not significantly reduce foraging by CPB females. After 96 h of the experiment, however, the mass of food eaten in the EO 0.2 DE 10 treatment was very similar to that in the EO 0.2 DE 0 treatment and did not differ significantly from the control (EO 0 DE 0). In the case of EO at concentrations of 0.5% and 1%, the effect of the DE addition did not result in significant changes in the foraging of CPB females. However, it should be emphasized that the mentioned EO doses caused a significant reduction in the foraging of CPB females compared to the control, persisting for 48 h of the experiment and, in the case of the EO 1 DE 0 and EO 1 DE 10 treatments, also significant after 96 h of the experiment.

**Figure 4:**
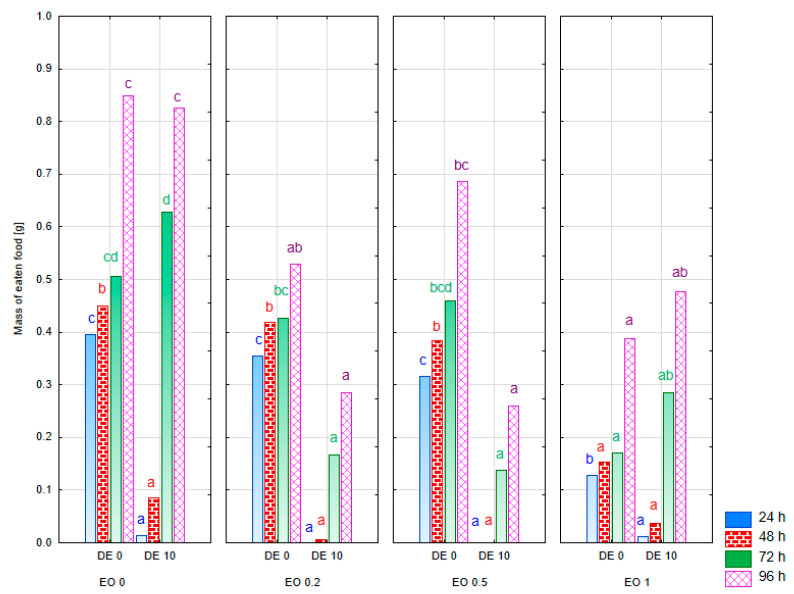
Mass of food eaten by one live male of *Leptinotarsa decemlineata* Say. [g] after the application of EO from *T. occidentalis* L. and diatomite (measurement in 24 h intervals from 24 to 96 h in relation to the initial mass of food each time; bars in the same color correspond to the respective observation dates; factors: EO × DE). For treatment descriptions, see Table 1. Different letters for values at specific time (the same color of font) mark statistically significant differences (*p* ≤ 0.05).

As with females, the addition of DE at a concentration of 10% to 0.2% EO from *T. occidentalis* in CPB males also resulted in a significant reduction in the mass of eaten food that was evident up to 72 h of the experiment (Figure 4, Table S4). In the case of EO 0.5%, the synergistic effect of DE addition was significant throughout the whole experiment. After 96 h, the mass of eaten food in the EO 0.5 DE 10 treatment was about 2.5 times lower than in the EO 0.5 DE 0 treatment. In the case of the EO 1% concentration, the synergistic effect of the DE addition was only visible in the first 24 h of the experiment. In contrast, DE alone reduced the foraging of CPB males during the first 48 h of the experiment.

**Figure 5:**
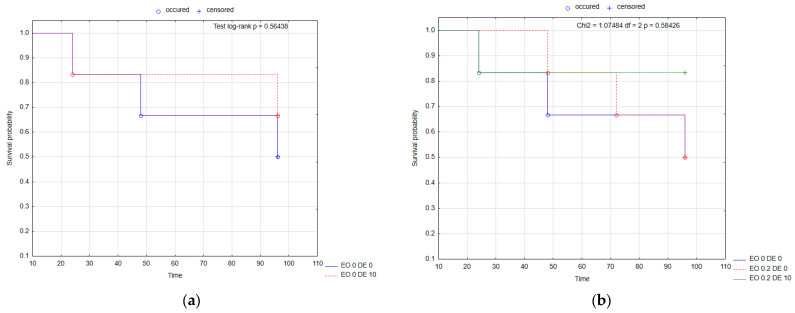

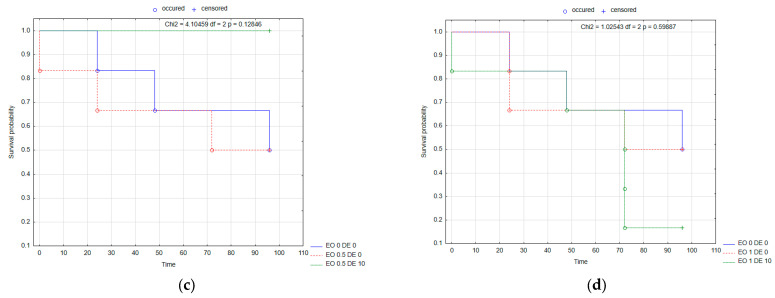
Kaplan–Meier survival curves of *L. decemlineata* females after the application of EO from *T. occidentalis* L. and diatomite: effect of DE alone (**a**); effect of EO in concentration 0.2% with and without DE (**b**); effect of EO in concentration 0.5% with and without DE (**c**); effect of EO in concentration 1% with and without DE (**d**). For treatment descriptions, see Table 1.

**Figure 6:**
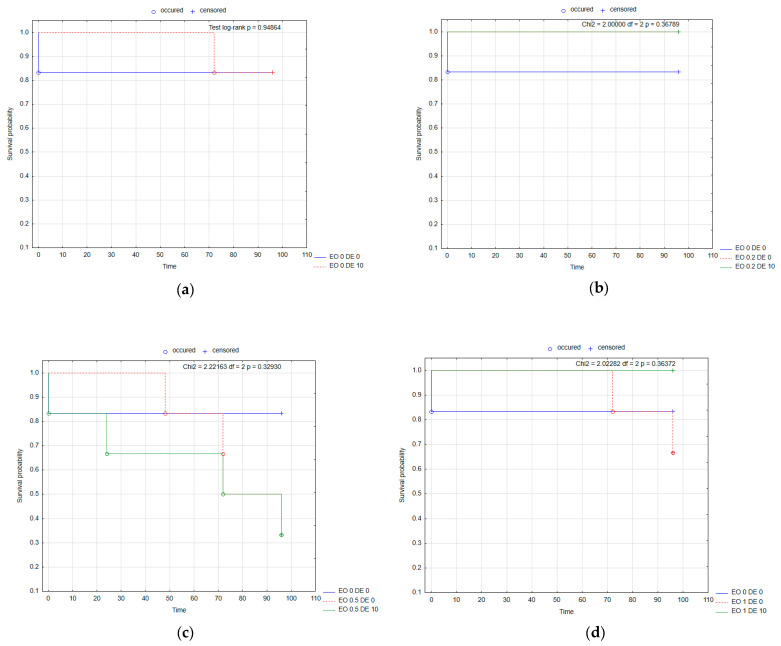
Kaplan–Meier survival curves of *L. decemlineata* males after the application of EO from *T. occidentalis* L. and diatomite: effect of DE alone (**a**); effect of EO in concentration 0.2% with and without DE (**b**); effect of EO in concentration 0.5% with and without DE (**c**); effect of EO in concentration 1% with and without DE (**d**). For treatment descriptions, see Table 1.

**Figure 7:**
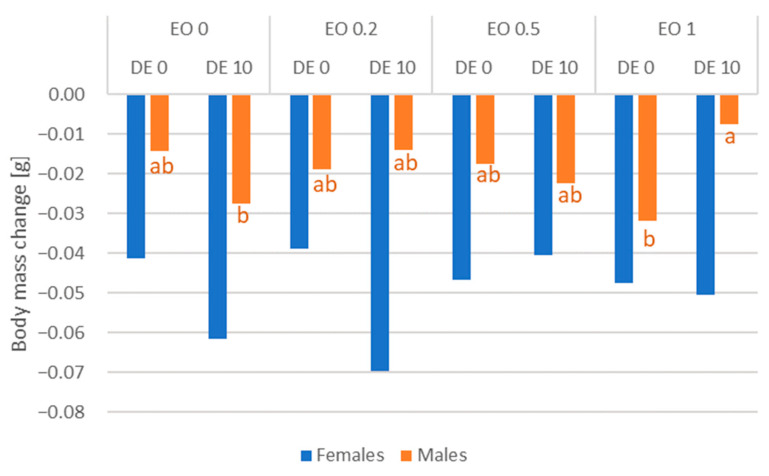
Body mass change of *L. decemlineata* females and males after 96 h after the application of EO from *T. occidentalis* L. and diatomite (factors: EO × DE). For treatment descriptions, see Table 1. Different letters for values for specific sex (the same color of font) mark statistically significant differences (*p* ≤ 0.05). Letters presented only if differences were significant.

The substances tested had no significant effect on mortality in female CPB (Figure 5, Table S5). Kaplan–Meier survival curves showed the lowest probability of survival for CPB females in the case of the EO 1 DE 10 mixture (Figure 5d). For males, some variation in mortality was recorded, but there was no effect of the addition of DE in any combination with EO compared to EO alone (except for EO 0.5% after 48 h, significant interaction of EO × DE according to ANOVA) (Table S6). The lowest probability of survival for CPB males was observed under the influence of the EO 0.5 DE 10 mixture (Figure 6). Changes in beetle weight were greater for females than for males. No statistically significant differences were found for females, while for males, there was less weight loss when DE 10% was added to EO 1% compared to the same treatment without DE addition (Figure 7, Table S7).

#### 2.2.2. Choice Experiment

**Figure 8:**
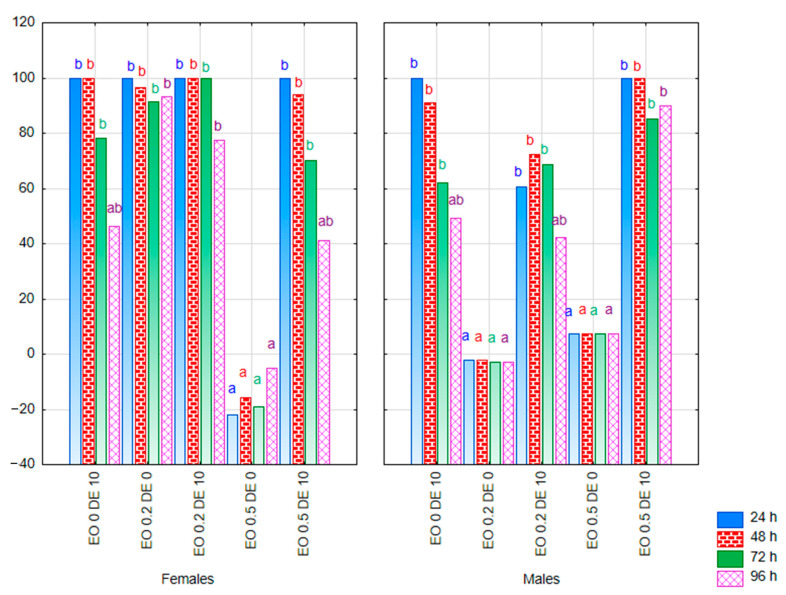
ADI value for females and males of *Leptinotarsa decemlineata* Say. [g] after the application of EO from *T. occidentalis* L. and diatomite (measurement in 24 h intervals from 24 to 96 h; bars in the same color correspond to the respective observation dates) in the choice experiment (factors: treatment × sex). For treatment descriptions, see Table 1. Different letters for values at specific time (the same color of font) mark statistically significant differences (*p* ≤ 0.05).

In the experiment, where CPB was offered food treated with additives or control food within a single dish, the absolute deterrence index (ADI) was used to assess the possible deterrent effect of the mixtures tested. The response of male and female CPB was compared. A two-way ANOVA showed a significant effect of the treatment factor on the ADI value at all observation dates. A significant interaction of both factors (treatment × sex) was also reported for most observation dates (Table S8; multivariate test results: treatment F = 4.91, *p* < 0.01; sex F = 2.41, *p* = 0.07; treatment × sex F = 3.39, *p* < 0.01). A significant reduction in the weight of food eaten by females was found when EO was used at a concentration of 0.2% and EO 0.2% in a mixture with DE at a concentration of 10% throughout the experiment (ADI value close to 100) (Figure 8). At the same time, a complete abandonment of foraging could be observed up to 72 h of the experiment on leaves treated with the EO 0.2 DE10 mixture. A similar effect of complete inhibition of feeding was also recorded at the higher dose of EO (0.5%) with the addition of DE, but only in the first 24 h of the experiment (ADI values = 100). It is noteworthy, however, that no deterrent effect was recorded when EO 0.5% was used alone (ADI value close to 0).

A slightly different response to the substances tested was observed in CPB males. There was no significant effect of EO at a concentration of 0.2% on the mass of food eaten (ADI close to 0). In this case, the addition of DE showed an enhancing effect, causing inhibition of foraging over a period of 72 h. In the case of the higher concentration of EO—0.5%, weak reduction in the mass of eaten food was observed under the influence of the EO used alone (ADI values of approximately 10) and very strong reduction in the case of EO 0.5 mixture with DE (ADI value from 85 to 100, depending on the time of observation).

When DE alone was used at a concentration of 10% the response of both sexes was similar: the complete inhibition of foraging for a period of 48 h for females and for 24 h for males was recorded (ADI = 100), as well as a significant reduction in foraging thereafter (ADI value > 45). However, the effect waned as time went on.

### 2.3. Sitona lineatus L.

#### 2.3.1. No-Choice Experiment

In the experiment where PLW females were offered the additive-treated food or the control food separately, the statistical analysis carried out for specific dates showed a significant effect of EO only (Table S9). There was no significant effect of the addition of DE, nor of the interaction of the two factors mentioned, at any of the observation dates analyzed. The results of multivariate tests for females were as follows: EO − F = 4.52, *p* < 0.001; DE − F = 2.27, *p* = 0.039; EO × DE − F = 3.14, *p* < 0.001. The first differences in the foraging intensity were found after 42 h of the experiment (Table 4). The significant effect of EO at a concentration of 0.2% persisted until 66 h. In this case, the addition of DE weakened the effectiveness of EO, so that there were no differences between the EO 0.2 DE 10 treatment and the control (EO 0 DE 0). The EO at 0.5% concentration resulted in a significant reduction in foraging of female PLW up to 78 h. Again, the addition of DE slightly attenuated the effect of EO. The highest concentration of EO—1% caused the strongest inhibition of female foraging—which was significant until the end of the experiment. In this case, the addition of DE also slightly weakened the effect of EO alone, but the effect compared to the control was statistically significant until the end of the experiment. After 114 h of the experiment, the surface area eaten in the treatment with EO at a concentration of 1% was about 4-fold lower than in the control, while in the same treatment but with the addition of DE, it was about 2.5-fold lower with respect to the control. Throughout the experiment, there was no significant limiting effect of DE alone at a concentration of 10% on the foraging of female PLW.

PLW males ate significantly less leaf blade than females (Table 5). An ANOVA performed for specific dates showed, as in females, a significant effect mainly of EO (Table S10). A significant effect of DE was also noted at some observation dates. However, there was no significant interaction of DE and EO. The results of multivariate tests for males were as follows: EO − F = 5.05, *p* < 0.001; DE − F = 3.78, *p* = 0.002; EO × DE − F = 4.23, *p* < 0.001. The use of DE alone at a concentration of 10% significantly reduced the foraging of PLW males (Table 5). After 114 h, the area eaten in the DE-supplemented treatment was about 2 times lower than in the control treatment. The effect of EO at the lowest concentration of 0.2%, as well as the same EO with added DE at a concentration of 10%, did not result in a significant reduction in the eaten leaf area. At the EO concentration of 0.5%, the addition of DE intensified the foraging-reducing effect, making it significant compared to the control most of the time, while EO alone at a concentration of 0.5% did not significantly reduce foraging. The strongest effect was obtained at an EO concentration of 1%. It was significant until the end of the experiment. After 114 h of the experiment, the area eaten in the EO 1% treatment was about 17 times lower than in the control, while in the same treatment with DE, it was nearly 6.5 times lower than in the control.

No case of PLW death under the influence of the substances tested was found.

#### 2.3.2. Choice Experiment

**Figure 9:**
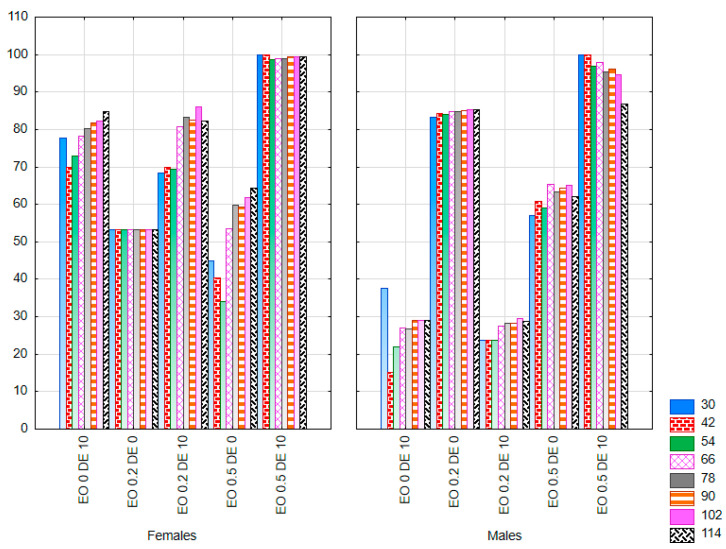
ADI value for females and males of *Sitona lineatus* L. after the application of EO from *T. occidentalis* L. and diatomite (measurement in 12 h intervals; bars in the same color correspond to the respective observation dates) in the choice experiment (factors: treatment × sex). For treatment descriptions, see Table 1. Differences not statistically significant (*p* ≤ 0.05).

In the experiment where females and males of PLW were offered food treated with additives or control food within a single dish, only treatment significantly influenced the ADI value (multivariate test results: treatment − F = 2.31, *p* < 0.01; sex − F = 1.87, *p* = 0.152; treatment × sex − F = 1.54, *p* = 0.08). However, the results for individual observation times separately showed no significant variation in this parameter under the influence of treatment or sex (Table S11). Due to the delay in the onset of foraging in some treatments on both control and treated leaves, a statistical analysis was only possible from 30 h of the experiment onwards. A significant reduction in the mass of food eaten was found when EO at a concentration of 0.5% was used in a mixture with DE at a concentration of 10% (Figure 9). Up to 42 h for females and 30 h for males of the experiment, a complete inhibition of foraging was recorded in this treatment (ADI = 100). In the later period, ADI values were also close to 100 in this treatment. The response of females and males to the DE alone was slightly different: the ADI value for males oscillated between 0 and 40 throughout the experiment, whereas, for females, it ranged between 69 and 84 most of the time, indicating a significant degree of foraging inhibition. In contrast, EO 0.2% applied alone showed a higher potential for inhibiting foraging in males than in females.

## 3. Discussion

### 3.1. Aphis fabae Scop.

One research hypothesis was that a mixture of EO + DE would cause higher herbivore mortality than its individual components separately (synergistic effect). In the case of BBA females, this was confirmed when a dose of 0.5% EO was used in combination with DE 5% and 10%, with better results obtained with DE 10%. For BBA nymphs, the enhancement of the insecticidal effect with the use of DE was obtained at the lowest EO dose of 0.2% and the DE dose of 10%. Thus, the response was dependent on both the dose and the developmental stage of the insect. The higher sensitivity to plant-based insecticides of BBA nymphs compared to wingless females was reported in the literature [43]. This is also confirmed by the LC_50_ values calculated in this experiment (clearly lower for nymphs during most of the observation period). It is interesting to note that, at the lower dose of EO—0.2%, the addition of DE weakened the effect of EO against wingless female aphids (antagonistic effect). Some protective effect against this stage was also observed at the highest dose of EO—i.e., 1%, but this was only evident during the first 42 h of the experiment. However, in the case of BBA nymphs, it was at the EO dose of 0.5% that a reduction in mortality under the influence of DE addition was recorded.

Both synergistic (increasing efficacy) and antagonistic (weakening the insecticidal effect) effects of mixtures of plant-derived substances and DE compared to the individual components separately were recorded for storage pests [22], and this was dependent on the pest species, the type of plant additive, and the type of DE product used. The synergistic effect is usually explained by the fact that desiccation due to cuticle damage (sorptive properties of DE) can be assisted by the antifeeding effect of EO. The antagonistic effect, on the other hand, can be explained by the absorption of EO by DE molecules, which results in a weakening of the action of both EO and DE (by reducing their lipid-binding capacity). In the present experiment, both an antagonistic effect (nymphs at a higher dose of EO; females at a lower dose of EO) and a synergistic effect (nymphs at a lower dose of EO; females at a higher dose of EO) were observed. A higher dose of EO may have inhibited the effect of desiccation from DE in nymphs. In the case of females, which may be more resistant to desiccation due to age and cuticle hardness, this mechanism of action of DE may have been less pronounced, while EO may have had a greater effect. In contrast, a lower dose of EO may not reduce desiccation from DE against nymphs as much. However, the sorption properties of DE may have reduced the effectiveness of the lower dose of EO against females. This may partly explain the observed differences in the response of the two life stages. However, confirmation that this particular type of effect manifests itself in a specific developmental stage of the same pest requires further research.

One of the objectives of the study was also to test whether the addition of DE would prolong and potentiate the effectiveness of EO. For BBA, this hypothesis was confirmed, as indicated by the calculated LC_50_ values. The effect was more pronounced in wingless females (lower LC_50_ values with DE addition starting at 54 h of the experiment compared to the treatment without DE addition) than in nymphs (lower LC_50_ values with DE 10 and DE 5 additions starting at 78 h and 114 h of the experiment, respectively, compared to the treatment without DE addition). The higher dose of DE resulted in a greater reduction in LC_50_ values. There are no data in the available literature on the use of mixtures of EO and DE against aphids or other pests with a piercing-sucking mouthparts. The use of DE (30 kg/ha) in combination with the wetting agent Wetcit (active ingredient orange oil/alcohol ethoxylate, 0.4% *v*/*v*) in Austrian elderberry orchards led to a clear reduction in oviposition (rates of infested berries and numbers of eggs per berry) by *Drosophila suzukii* Matsumura as compared to the untreated control plot (in the initial period at a level almost equal to those with the insecticide SpinTor) [23]. The authors also demonstrated the importance of thorough and continuous coverage of berries with the mixture of the mentioned substances for effective control of the pest. However, such a clear effect of the mentioned mixture on the quantity of instars and pupae in berries on the trees was not reported. Furthermore, DE itself was not tested in this experiment.

The use of DE alone in the present study showed an increase in mortality of BBA wingless females to a maximum of about 24% and this effect was externalized earlier at the 10% dose. Nymphs also showed an increase in mortality of about 25% at the DE 5% dose and 21–31% at the DE 10% dose (depending on the observation date), with the higher dose showing a significant effect 36 h earlier. There is no information on the use of DE against BBA in the available literature. DE applied in the form of dust under field conditions (foliar dose of 150 kg DE/ha and higher doses) in wheat crops reduced the population of *Rhopalosiphum padi* aphid only for the initial two days. At the same time, wheat plants coated with DE dust showed reduced chlorophyll content [19]. When DE was applied to the soil in laboratory tests, a reduction in fecundity, adult longevity, and total development duration of this aphid was recorded with increasing doses of DE. In contrast, under field conditions, such use of DE did not result in significant differences compared to the control. The use of a kaolin-based formulation (Sepidan (R)) along with diatomaceous earth (Sayan (R)) resulted in a 50% reduction in vine cicada *Psalmocharias alhageos* (Kolenati 1857) egg laying in comparison to untreated control [44]. Studies on the reduction in another pest with a piercing-sucking mouth apparatus, African citrus psyllid *Trioza erytreae* (Del Guercio), using i.a. diatomaceous earth in semi-field and field trials [45] showed its high efficacy, but only in semi-field conditions.

Summarizing the data at the end of the experiments, i.e., after 114 h, revealed that EO at a concentration of 1%, regardless of the addition of DE, caused 100% mortality of both nymphs and wingless female aphids. However, with the aim of reducing the concentration of EO, due to its potential phytotoxicity and the reduction in production costs, as well as the possible threat to beneficial fauna [32], the best insecticidal effects were obtained with EO 0.5% with DE 10% added for wingless females (more than 3-fold reduction in the survival rate). Even with the antagonistic effect of DE taken into account, the same combination resulted in a more than 4.6-fold reduction in the survival of BBA nymphs. In comparison, an EO dose of 0.2% with 10% DE resulted in a 2.5-fold reduction in nymph survival and an approximately 2-fold reduction in the survival of wingless females.

### 3.2. Leptinotarsa decemlineata Say.

The hypothesis that the EO + DE mixture would show a stronger and more sustained foraging inhibitory effect against CPB than the use of EO or DE alone was confirmed when a low concentration of EO, i.e., 0.2%, was used, with the effect being evident for 48 h for females and 72 h for males from the start of the experiment. In the case of males, a synergistic effect was also observed at a dose of 0.5% EO and the addition of DE at a concentration of 10% for the entire duration of the experiment and at a dose of 1% EO, but only for the initial 24 h of the experiment. In conclusion, in the case of females, all mixtures and their components separately (except EO at a concentration of 0.2%) significantly reduced beetle foraging, but the effect was short-lasting (up to 48 h), while in the case of males, the most favorable effects were obtained with a mixture of EO 0.5 DE 10 (after 96 h of experiment, more than 3 times lower weight of eaten food than in the control). At the same time, EO 0.5% or DE used alone did not significantly inhibit foraging compared to the control. There was no significant antagonistic effect of DE in any of the cases studied.

When adult CPB were given a choice between the control food and food treated with the test substances alone or in mixtures (choice experiment), it was observed that the beetles chose less frequently the food treated with EO alone (only EO 0.2% in females), DE alone, or their mixtures. Already, the use of EO at a concentration of 0.2% in a mixture with DE 10% resulted in the complete cessation of feeding by females on the leaves treated in this way for up to 72 h of the experiment (ADI = 100). A similar effect in the case of males (the complete inhibition of feeding up to 48 h of the experiment) was recorded at a higher dose of EO (0.5%) in a mixture with DE 10%. In general, the addition of DE resulted in higher ADI values compared to EO used alone in males for both EO concentrations tested and in females for the higher concentration (0.5%). At the same time, no antagonistic effect of DE was observed.

The available literature on the effect of mixtures of DE with EO on beetle pests relates to storage pests, with reports of both synergistic and antagonistic effects [16,46,47,48]. DE in combination with botanicals (essential oil lavender, corn oil, and bay leaf dust) showed higher efficacy against the storage pests *Sitophilus oryzae* (L.), *Rhyzopertha dominica* (F.), and *Tribolium castaneum* (Herbst) in seed wheat and barley than DE alone [46]. An increase in the insecticidal activity of DE against *T. castaneum* was achieved via the addition of EO from *Thymus capitatus* (L.) [47], as well as via the addition of odorless garlic powder against *T. confusum* du Val. [16]. However, studies on the individual and combined effect of plant-based essential oil alpha-pinene and DE against stored product beetles showed a weakening of the effect when the mixture was used compared to each component used separately [48].

DE used alone in the no-choice experiment in the present study contributed to a reduction in the weight of food eaten by adult CPB compared to the control only in the initial 48 h of the experiment. Thereafter, the beetles made up for the initial foraging discouragement and, after 72 h, the weight of food eaten in the DE-supplemented treatment did not differ from the control. In the choice experiment, on the other hand, there was a complete inhibition of foraging by CPB females for 48 h and by males for 24 h (ADI = 100), as well as a significant reduction in foraging thereafter (ADI value > 45).

There are no data in the available literature on the use of DE as an aqueous solution against the potato beetle. DE used as dust against larvae and adult CPB (application on leaves and insects) showed some limited insecticidal effectivenes (less than 20% in 7 days of exposure) against adult CPB. When DE was applied only to leaves or only to insects, there was no significant effectiveness [49]. On the other hand, the authors noted a reduction in the feeding of adult CPB when DE dust was added to leaves and to beetles (defoliation of 27% on the 7th day of exposure, against 100% in the control). When DE dust was applied to leaves but not to beetles, the effect was much weaker (defoliation of about 70% on the 7th day of exposure, against 100% in the control). In contrast, when DE dust was applied only to beetles, the effect was insignificant. In the aforementioned experiment, the sex of adult CPB was not differentiated. According to the literature [18], the effectiveness of DE in the form of an aqueous solution is weaker than that used in the form of dust, which may partly explain the rather poor foraging inhibition results obtained in the present experiment.

The mixtures of substances tested did not significantly increase the mortality of adult CPB, nor did they affect body weight during the experiment.

### 3.3. Sitona lineatus L.

In contrast to CPB, in the case of PLW, there was generally no effect of increasing the effectiveness of EO by the addition of DE. The reported few instances of a synergistic effect when mixtures were used, compared to the components used separately, were dose- and sex-dependent. The mixture of EO + DE (irrespective of the dose of EO) in the no-choice experiment did not show a stronger effect than EO at the appropriate dose used alone in reducing the foraging of female PLW. However, the effect of the mixture of DE with EO at the highest concentration (1%) was stronger than DE alone, which did not significantly affect the foraging of females. In the case of males, there was a synergistic effect of DE, potentiating the effect of EO at a concentration of 0.5% (more than 2 times less leaf blade area eaten in the treatment with DE compared to the treatment without DE).

In contrast, in the choice experiment, where weaker doses of EO (0.2% and 0.5%) were tested in the context of potential use to discourage foraging, a significant inhibition of PLW foraging was recorded for the mixture with the maximum content of substances tested, i.e., EO 0.5 DE10 (ADI close to 100). When the beetles have a choice between the control food and additive-treated food, the observed effects should be more noticeable than when the insects must either eat the offered food (despite being treated with additives) or starve. And this was confirmed in the case of females, as in the no-choice experiment, the EO 0.5 DE 10 treatment was not significantly different from the control in terms of eaten leaf area, except for the short initial period (42 and 54 h of the experiment).

There is no information in the available literature on the different sex responses of *Sitona* beetles to diatomite, and most of the available data on sex differences relate to storage pests and, as in the present experiment, indicate that males are more sensitive. In an experiment where adult bean weevils (*Acanthoscelides obtectus* [Say]) were exposed to eight different DE dosages (400–4000 mg/kg), DE dust was more lethal to males than to female weevils within shorter exposure periods at low DE dosages (<2400 mg/kg). However, LD_99_ values did not differ between the sexes [50]. In contrast, the mortality of *T. castaneum* adults exposed for 7 days to hard red winter wheat treated with 0.25 g/kg of INSECTO (commercial diatomite) was similar between the sexes [51]. In the present experiment, PLW males were clearly more susceptible to diatomite in no-choice experiment, and in addition, in their case, synergism in the application of the substances tested in the mixture was noted, but only at the EO dose of 0.5%. However, in the choice experiment, the ADI value using diatomite alone was relatively low for males (0–40). Most of the literature on the effect of sex on the response to plant-based substances used as insecticides also indicates that males are more sensitive [52,53,54]. This is explained by differences in body size, cuticule composition, and physiology, which may affect the ability to cope with toxic substances.

## 4. Materials and Methods

### 4.1. Experimental Design

**Figure 10:**
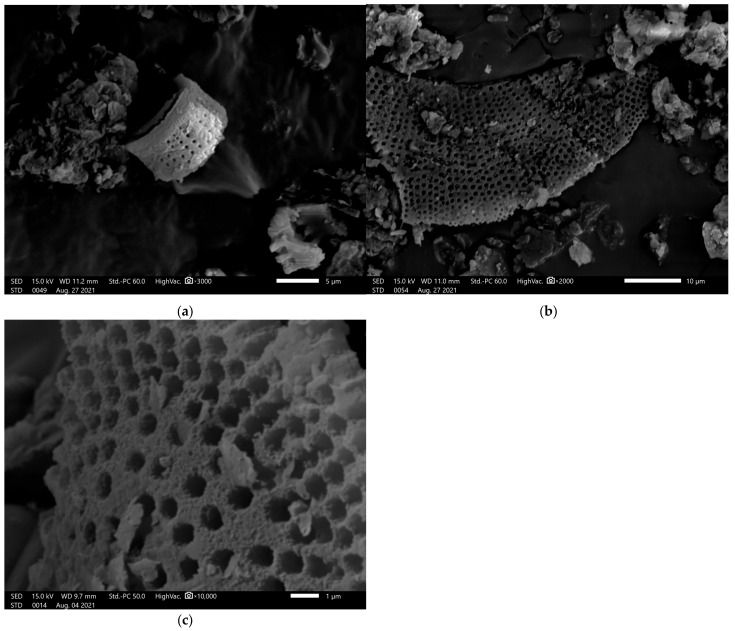
SEM images of diatomite, different shapes (**a**,**b**) and magnification (**c**).

Diatomite (Table 6) was supplied by the Specialized Mining Company ‘Górtech’ Sp. z o. o. The material (Figure 10 a–c) was extracted from a deposit located in Bircza, Poland (49°43′16.9″ N; 22°18′25.4″ E). The diatomite was subjected to a calcination process (chamber furnace, 750 °C, 0.5 h). A fraction of 5–100 µm diatomite was included in the study.

EO from *T. occidentalis* was extracted via hydrodistillation using the Clevenger apparatus from leaves collected in June 2022. The chemical composition of the EO used was reported in an earlier publication [32]. Among the 40 components identified (which was 99.1% of the total compounds in this oil), the main constituents were: α-thujone (38.5%), sabinene (12.9%), fenchone (9.3%), terpinen-4-ol (7.3%), β-thujone (4.9%), and bornyl acetate (4.3%). To obtain the basic solution of EO (10%), 96% ethanol was used. Final dilutions of EO were obtained by adding the appropriate amount of redistilled water to the base dilution. In turn, the mixtures used in the experiment were prepared by adding the appropriate amount of diatomite to the previously prepared EO solution and mixing them thoroughly. The following doses of EO were used in the experiment: 0.2%, 0.5%, and 1%, and two doses of diatomite: 5% and 10%. Redistilled water was used as a control.

The host plant leaves of a given pest, selected for size and age, were immersed in the prepared solutions (before each leaf was immersed, the solution was stirred vigorously to obtain a homogeneous suspension) and then placed in Petri dishes (diameter 9 cm for no-choice experiments and 14 cm for choice experiments, with ventilation), lined with moist filter paper. The insects used in the experiments were obtained from a culture conducted for this purpose at the Department of Microbiology and Biomonitoring of the University of Agriculture in Krakow. The experiments were conducted in the laboratory (24 °C ± 1°, daylight) in six replicates.

### 4.2. Aphis fabae Scop.

One leaf of mock-orange (*Philadelphus coronarius* L.), prepared in the way described above, was placed per dish, along with 10 individuals (wingless females or 6-day-old nymphs). The experiment included the following treatments: control—redistilled water (EO 0 DE 0), EO 0.2 − EO in the concentration of 0.2% (EO 0.2 DE 0), EO 0.2 + DE 5 − EO in the concentration of 0.2% + diatomite in the concentration of 5% (EO 0.2 DE 5), EO 0.2 + DE 10 − EO in the concentration of 0.2% + diatomite in the concentration of 10% (EO 0.2 DE 10), EO 0.5 − EO in the concentration of 0.5% (EO 0.5 DE 0), EO 0.5 + DE 5 − EO in the concentration of 0.5% + diatomite in the concentration of 5% (EO 0.5 DE 5), EO 0.5 + DE 10 − EO in the concentration of 0.5% + diatomite in the concentration of 10% (EO 0.5 DE 10), EO 1 − EO in the concentration of 1.0% (EO 1 DE 0), EO 1 + DE 5 − EO in the concentration of 1.0% + diatomite in the concentration of 5% (EO 1 DE 5), EO 1 + DE 10 − EO in the concentration of 1.0% + diatomite in the concentration of 10% (EO 1 DE 10), DE 5 − diatomite in the concentration of 5% (EO 0 DE 5), DE 10–diatomite in the concentration of 10% (EO 0 DE 10). The mortality of aphids was measured ten times, first after 6 h and then every 12 h.

### 4.3. Leptinotarsa decemlineata Say.

Two types of experiments were carried out: no-choice and choice experiments. The no-choice experiment was performed to evaluate the effect of the substances tested on foraging intensity, mortality, and body weight changes of CPB imagoes. For this purpose, one leaf of *Solanum tuberosum* L., the Bella rosa cultivar, treated with the appropriate solution was placed in a 9 cm diameter dish. One adult (female or male) was then placed in each dish. The experiment included the following treatments: control—redistilled water (EO 0 DE 0), EO 0.2 − EO in the concentration of 0.2% (EO 0.2 DE 0), EO 0.2 + DE 10 − EO in the concentration of 0.2% + diatomite in the concentration of 10% (EO 0.2 DE 10), EO 0.5 − EO in the concentration of 0.5% (EO 0.5 DE 0), EO 0.5 + DE 10 − EO in the concentration of 0.5% + diatomite in the concentration of 10% (EO 0.5 DE 10), EO 1 − EO in the concentration of 1.0% (EO 1 DE 0), EO 1 + DE 10 − EO in the concentration of 1.0% + diatomite in the concentration of 10% (EO 1 DE 10), DE 10 − diatomite in the concentration of 10% (EO 0 DE 10). Due to the greater resistance of CPB compared to aphids, the lower dose of diatomite was abandoned. Mass of eaten food at specific time intervals of the experiment (every 24 h) was measured, as well as the mortality of CPB. Body weight changes were also measured after 96 h of the experiment (each individual was weighed separately at the beginning and end of the experiment).

In the second type of experiment (choice experiment), conducted to evaluate the repellent effect, two host plant leaves were placed in a 14 cm diameter dish: one serving as a control—treated with redistilled water—and the other treated with the appropriate solution. One adult (female or male) was then placed in each dish. Mass of eaten food (separately for control and treated leaves) at specific time intervals of the experiment (every 24 h) was measured. The following treatments were tested: EO 0.2 − EO in the concentration of 0.2% (EO 0.2 DE 0), EO 0.2 + DE 10 − EO in the concentration of 0.2% + diatomite in the concentration of 10% (EO 0.2 DE 10), EO 0.5 − EO in the concentration of 0.5% (EO 0.5 DE 0), EO 0.5 + DE 10 − EO in the concentration of 0.5% + diatomite in the concentration of 10% (EO 0.5 DE 10), DE 10—diatomite in the concentration of 10% (EO 0 DE 10).

### 4.4. Sitona lineatus L.

The experiments (both no-choice and choice) were carried out analogously to CPB. *Vicia faba* L., Bartek cultivar, was used as the host plant. Experiments were conducted separately for males and females. The leaf area eaten by pea leaf weevil beetles was measured, as well as the mortality of the insects.

### 4.5. Statistical Analysis

The data were pre-checked for normality (Shapiro–Wilk test with Lilliefors correction) and equality of variance (Levene’s test). The significance of differences between the mean values in the case of aphids and no-choice experiments for CPB and PLW was tested via a two-way ANOVA (STATISTICA 13.0 software, factors: EO and DE). Then, a post hoc LSD test was used at *p* ≤ 0.05. LC_50_ values for BBA were calculated according to Finney [55]. Kaplan–Meier survival curves were prepared using the STATISTICA 13.0 software. In the choice experiments (CPB and PLW), the deterrent effect was assessed using the calculation of absolute deterrence index (ADI = [(K − T)/(K + T)] × 100, where K is the mass ([g] for CPB) or area ([mm^2^] for PLW) of the control leaf consumed by the pest, and T is the mass ([g] for CPB) or area ([mm^2^] for PLW) of the treated leaf consumed by the pest. In this case, a two-way ANOVA was used to compare the effect of the treatments used and gender (factors: treatment (mixtures of EO + DE, as well as EO or DE alone as one factor) and sex (females or males as the second factor).

## 5. Conclusions

1. The improvement in effectiveness achieved using a mixture of EO + DE instead of the individual components separately against BBA was dependent on both the dose of the components and the developmental stage of the BBA. The addition of DE enhanced the effect of EO at a concentration of 0.5% against wingless females and the effect of EO at a concentration of 0.2% against nymphs, with better effects obtained at a dose of 10% of DE. Despite the observed antagonistic effects of DE (DE 5% for EO 0.2% and EO 1% treatments against wingless females and DE 5% and DE 10% for EO 0.5% against nymphs), the calculated LC_50_ values indicate a prolongation of the insecticidal effect of EO from *T. occidentalis* against both developmental stages of BBA due to the addition of DE with a stronger effect at the higher DE dose—10%. This effect is revealed with a delay of 2–3 days. The mixtures tested, in general, did not increase the mortality of CPB and PLW in comparison to individual components alone.
2. The effect of enhancing CPB foraging inhibition by using a mixture of EO + DE instead of the individual components separately was obtained at a concentration of 0.2% EO (both females and males of CPB) and 0.5% EO (males) in the no-choice experiments. In the choice experiments, the mixtures EO + DE with both 0.2% and 0.5% EO concentrations resulted in a significant reduction in CPB foraging, with a synergistic DE effect recorded for EO 0.2% for males and for EO 0.5% for both males and females.
3. In the case of PLW, a significant strengthening effect of EO via the addition of DE at a dose of 10% in the no-choice experiment was obtained only in the case of males, at an EO concentration of 0.5%, while when the beetles had a choice the synergistic effect of a mixture of EO 0.5% and DE 10% was also apparent in the case of females.
4. No significant antagonistic effect of DE in mixtures with EO against CPB and PLW foraging was demonstrated.

In conclusion, the use of DE mixtures with EO from *T. occidentalis* appears to be a promising strategy, but it requires further research in the context of safety for non-target entomofauna and the protected plant (phytotoxicity effect). The results obtained concerning the foraging inhibition of CPB and PLW, as well as those on the mortality of BBA at relatively low doses of EO in the mixture with DE, support the idea of not using doses of EO higher than 0.5%.

## Figures and Tables

**Table 1:** Survival of *Aphis fabae* Scop. wingless females after the application of EO from *T. occidentalis* L. and diatomite. EO 0—redistilled water, EO 0.2—EO in the concentration of 0.2%, EO 0.5—EO in the concentration of 0.5%, EO 1—EO in the concentration of 1.0%, DE 5—diatomite in the concentration of 5%, DE 10—diatomite in the concentration of 10%.

Hours	EO 0			EO 0.2			EO 0.5			EO 1		
	DE 0	DE 5	DE 10	DE 0	DE 5	DE 10	DE 0	DE 5	DE 10	DE 0	DE 5	DE 10
6	100.00 (±0.00) c*	100.00 (±0.00) c	100.00 (±0.00) c	100.00 (±0.00) c	100.00 (±0.00) c	100.00 (±0.00) c	100.00 (±0.00) c	100.00 (±0.00) c	98.00 (±1.63) c	0.00 (±0.00) a	31.67 (±7.03) b	23.33 (±10.54) b
18	100.00 (±0.00) c	100.00 (±0.00) c	100.00 (±0.00) c	98.00 (±1.63) c	96.00 (±2.00) c	98.00 (±1.63) c	100.00 (±0.00) c	100.00 (±0.00) c	98.00 (±1.63) c	0.00 (±0.00) a	31.67 (±7.03) b	23.33 (±10.54) b
30	100.00 (±0.00) c	100.00 (±0.00) c	98.00 (±1.63) c	94.00 (±3.27) c	96.00 (±2.00) c	96.00 (±2.00) c	100.00 (±0.00) c	100.00 (±0.00) c	94.00 (±3.27) c	0.00 (±0.00) a	21.67 (±5.43) b	23.33 (±10.54) b
42	100.00 (±0.00) c	100.00 (±0.00) c	98.00 (±1.63) c	92.00 (±3.06) c	92.00 (±1.63) c	92.00 (±1.63) c	100.00 (±0.00) c	90.00 (±8.16) c	90.00 (±6.32) c	0.00 (±0.00) a	21.67 (±5.43) b	23.33 (±10.54) b
54	98.00 (±1.63) e	90.00 (±2.58) de	86.00 (±3.27) d	84.00 (±2.00) cd	90.00 (±2.58) de	86.00 (±2.00) d	92.00 (±1.63) de	75.00 (±5.48) bc	70.00 (±7.30) b	0.00 (±0.00) a	8.33 (±3.07) a	5.00 (±3.42) a
66	96.00 (±2.00) e	78.00 (±5.42) d	74.00 (±3.27) cd	74.00 (±4.90) cd	76.18 (±4.27) d	76.00 (±4.16) d	84.00 (±3.27) d	63.00 (±7.70) c	50.00 (±5.16) b	0.00 (±0.00) a	1.67 (±1.67) a	3.33 (±2.11) a
78	90.00 (±2.58) f	71.56 (±4.65) e	66.00 (±4.16) d	56.00 (±7.12) cd	70.36 (±3.83) d	64.00 (±3.27) de	72.00 (±4.76) e	49.00 (±10.68) bc	36.00 (±7.12) b	0.00 (±0.00) a	1.67 (±1.67) a	0.00 (±0.00) a
90	74.00 (±3.27) f	61.56 (±5.18) de	62.00 (±4.76) de	48.00 (±7.92) cd	64.36 (±5.72) e	54.00 (±2.00) de	66.00 (±5.54) e	36.50 (±8.15) bc	24.00 (±6.63) b	0.00 (±0.00) a	0.00 (±0.00) a	0.00 (±0.00) a
102	68.00 (±3.06) g	48.89 (±4.56) def	56.00 (±6.11) efg	40.00 (±8.94) bcd	57.09 (±5.06) efg	46.00 (±3.27) cde	66.00 (±5.54) fg	32.50 (±6.77) bc	26.00 (±7.12) b	0.00 (±0.00) a	0.00 (±0.00) a	0.00 (±0.00) a
114	58.00 (±4.00) e	44.89 (±3.16) de	48.00 (±4.76) de	30.00 (±8.56) bc	39.09 (±6.65) cd	28.00 (±4.76) bc	54.00 (±8.41) e	20.50 (±5.02) b	18.00 (±4.76) b	0.00 (±0.00) a	0.00 (±0.00) a	0.00 (±0.00) a

* Different letters in lines mark statistically significant differences (*p* ≤ 0.05).

**Table 2:** Survival of *Aphis fabae* Scop. nymphs after the application of EO from *T. occidentalis* L. and diatomite. For treatment descriptions, see Table 1.

Hours	EO 0			EO 0.2			EO 0.5			EO 1		
	DE 0	DE 5	DE 10	DE 0	DE 5	DE 10	DE 0	DE 5	DE 10	DE 0	DE 5	DE 10
6	100.00 (±0.00) c *	100.00 (±0.00) c	100.00 (±0.00) c	100.00 (±0.00) c	100.00 (±0.00) c	100.00 (±0.00) c	100.00 (±0.00) c	97.78 (±1.81) b	100.00 (±0.00) c	0.00 (±0.00) a	0.00 (±0.00) a	0.00 (±0.00) a
18	100.00 (±0.00) e	100.00 (±0.00) e	98.00 (±1.63) de	97.78 (±1.81) de	96.00 (±2.00) d	100.00 (±0.00) e	45.45 (±11.00) b	79.56 (±5.78) c	87.14 (±4.75) cd	0.00 (±0.00) a	0.00 (±0.00) a	0.00 (±0.00) a
30	100.00 (±0.00) e	98.00 (±1.63) e	98.00 (±1.63) e	93.78 (±2.08) e	88.00 (±6.00) de	96.00 (±2.00) e	40.45 (±10.87) b	73.56 (±7.06) c	77.14 (±7.73) cd	0.00 (±0.00) a	0.00 (±0.00) a	0.00 (±0.00) a
42	100.00 (±0.00) f	95.78 (±2.12) ef	95.78 (±2.12) ef	93.78 (±2.08) ef	81.78 (±6.98) de	92.00 (±3.06) ef	35.45 (±11.05) b	55.70 (±7.59) c	69.14 (±11.61) cd	0.00 (±0.00) a	0.00 (±0.00) a	0.00 (±0.00) a
54	100.00 (±0.00) e	93.78 (±3.32) de	95.78 (±2.12) e	93.78 (±2.08) de	77.78 (±6.96) d	88.00 (±3.06) de	32.95 (±11.03) b	47.25 (±7.32) bc	52.64 (±11.61) c	0.00 (±0.00) a	0.00 (±0.00) a	0.00 (±0.00) a
66	94.00 (±2.00) d	90.92 (±3.24) cd	83.56 (±5.60) cd	85.56 (±3.42) cd	75.78 (±7.51) c	78.00 (±5.42) cd	25.45 (±11.98) b	35.43 (±9.64) b	38.79 (±9.81) b	0.00 (±0.00) a	0.00 (±0.00) a	0.00 (±0.00) a
78	94.00 (±2.00) f	84.48 (±1.97) ef	73.11 (±8.41) de	83.33 (±4.55) ef	73.78 (±7.50) de	66.00 (±5.54) d	13.64 (±8.62) ab	29.39 (±9.20) bc	31.07 (±8.59) c	0.00 (±0.00) a	0.00 (±0.00) a	0.00 (±0.00) a
90	90.00 (±4.47) e	80.25 (±4.04) de	63.11 (±6.40) c	79.33 (±5.40) de	67.78 (±9.37) cd	54.00 (±6.63) c	4.55 (±2.87) a	23.17 (±9.59) b	25.71 (±6.61) b	0.00 (±0.00) a	0.00 (±0.00) a	0.00 (±0.00) a
102	88.00 (±6.00) f	73.59 (±8.73) ef	57.11 (±8.44) de	69.33 (±2.64) e	61.56 (±10.01) de	46.00 (±6.63) d	2.27 (±1.44) ab	17.54 (±6.89) bc	23.71 (±7.08) c	0.00 (±0.00) a	0.00 (±0.00) a	0.00 (±0.00) a
114	81.78 (±4.70) f	66.73 (±7.85) e	52.89 (±8.17) de	59.33 (±4.97) de	50.44 (±4.86) d	32.00 (±4.76) c	2.27 (±1.44) a	9.49 (±5.76) ab	17.71 (±7.85) bc	0.00 (±0.00) a	0.00 (±0.00) a	0.00 (±0.00) a

* Different letters in lines mark statistically significant differences (*p* ≤ 0.05).

**Table 3:** The LC_50_ values for *T. occidentalis* EO recorded against wingless females and nymphs of *Aphis fabae* Scop. selected hours after treatment without diatomite (EO) and with two doses of diatomite (5%—DE 5 and 10%—DE 10).

Life Stage	Hours	EO			EO + DE 5			EO + DE 10		
		LC_50_ (%)	Slope *	(X^2^) **	LC_50_ (%)	Slope *	(X^2^) **	LC_50_ (%)	Slope *	(X^2^) **
Wingless females	18	0.7181	7.8505	355.3289	0.9022	3.5197	34.6348	0.8638	4.1740	45.4694
	30	0.7039	5.6527	161.9125	0.8513	3.9660	40.1321	0.8325	3.3057	33.1705
	42	0.6978	5.0852	107.8235	0.7879	2.7300	25.1243	0.8022	2.6686	33.0724
	54	0.6398	3.5678	40.0723	0.6376	3.0195	12.4940	0.5851	2.9668	16.8741
	66	0.5763	2.7490	41.6216	0.5001	2.7469	20.3934	0.4573	2.6327	10.7502
	78	0.4493	1.9822	43.5270	0.4261	2.5742	22.2935	0.3390	2.7539	12.2589
	90	0.3741	1.7494	44.3555	0.3467	2.7643	17.2943	0.2478	2.7629	10.5112
	102	0.3142	1.5142	52.8055	0.2915	2.5395	13.8415	0.1992	2.3294	14.1463
	114	0.1402	1.2991	51.7609	0.1328	2.2953	13.5444	0.0023	1.9314	12.5355
Nymphs	18	0.4886	6.8461	20.0916	0.6083	5.2449	19.3283	-	-	-
	30	0.4645	5.3497	18.0705	0.5740	3.7539	36.5791	0.5885	5.2016	18.2798
	42	0.4489	5.6098	19.2999	0.4847	3.3085	24.8664	0.5503	4.3164	25.6042
	54	0.4115	4.9089	23.5833	0.4393	3.1616	20.5897	0.4281	3.7787	26.8713
	66	0.3912	4.7919	27.0346	0.3862	3.3608	24.4905	0.3938	3.5934	17.9112
	78	0.3441	5.6946	23.5351	0.3569	3.4783	23.0014	0.3226	3.1379	16.2661
	90	0.2942	7.2231	11.0381	0.3117	3.4200	29.4628	0.2478	2.7635	14.6038
	102	0.2545	7.5146	3.7803	0.2668	3.4902	23.3281	0.1901	2.5221	16.8019
	114	0.2275	6.7729	5.8554	0.2021	3.8054	15.4086	0.0569	2.2872	20.1405

* Slope of the regression line, ** Chi-square value (16 df), *p* < 0.05.

**Table 4:** Leaf area eaten by one live female of *Sitona lineatus* L. [mm^2^] after the application of EO from *T. occidentalis* L. and diatomite. For treatment descriptions, see Table 1.

Hours	EO 0		EO 0.2		EO 0.5		EO 1	
	DE 0	DE 10	DE 0	DE 10	DE 0	DE 10	DE 0	DE 10
6	0.39 (±0.27) a*	0.00 (±0.00) a	0.00 (±0.00) a	0.00 (±0.00) a	0.00 (±0.00) a	0.00 (±0.00) a	0.00 (±0.00) a	0.00 (±0.00) a
18	43.58 (±12.25) a	46.59 (±15.12) a	1.05 (±0.52) a	43.54 (±14.99) a	26.09 (±14.69) a	36.48 (±21.23) a	15.66 (±8.47) a	20.70 (±13.03) a
30	58.11 (±14.99) a	75.91 (±14.78) a	52.65 (±13.59) a	52.48 (±14.38) a	36.68 (±13.94) a	58.45 (±20.15) a	22.37 (±8.03) a	28.65 (±13.03) a
42	128.72 (±27.81) c	106.39 (±31.14) bc	56.58 (±12.31) ab	77.90 (±17.84) abc	43.94 (±12.70) a	63.26 (±17.65) ab	33.81 (±11.28) a	35.81 (±15.32) a
54	132.53 (±28.34) c	126.34 (±36.25) bc	60.90 (±10.48) a	84.22 (±16.64) abc	51.14 (±13.29) a	69.54 (±16.38) ab	35.54 (±10.99) a	40.81 (±14.16) a
66	143.71 (±26.30) c	128.96 (±36.89) c	70.03 (±8.23) ab	97.73 (±13.89) bc	63.11 (±11.69) ab	97.74 (±15.05) bc	36.32 (±11.04) a	43.65 (±13.79) ab
78	146.85 (±26.87) d	132.61 (±35.43) cd	95.05 (±13.13) bcd	100.57 (±14.05) bcd	88.52 (±11.09) abc	107.26 (±16.34) bcd	36.71 (±11.07) a	56.21 (±13.21) ab
90	158.43 (±26.85) c	146.21 (±33.37) c	131.46 (±16.96) c	115.26 (±13.75) c	108.87 (±13.61) bc	129.80 (±11.60) c	38.67 (±10.63) a	58.96 (±12.75) ab
102	164.32 (±27.90) c	154.19 (±34.90) c	139.57 (±16.56) c	122.03 (±15.84) c	108.87 (±13.26) bc	138.82 (±11.76) c	40.24 (±10.25) a	59.35 (±12.81) ab
114	164.32 (±27.90) c	154.84 (±34.65) c	158.61 (±19.74) c	124.58 (±16.15) bc	121.86 (±15.80) bc	158.14 (±8.46) c	40.24 (±10.25) a	67.99 (±15.25) ab

* Different letters in lines mark statistically significant differences (*p* ≤ 0.05).

**Table 5:** Leaf area eaten by one live male of *Sitona lineatus* L. [mm^2^] after the application of EO from *T. occidentalis* L. and diatomite. For treatment descriptions, see Table 1.

Hours	EO 0		EO 0.2		EO 0.5		EO 1	
	DE 0	DE 10	DE 0	DE 10	DE 0	DE 10	DE 0	DE 10
6	1.67 (±0.54) a*	0.39 (±0.27) a	6.28 (±4.29) a	0.00 (±0.00) a	0.00 (±0.00) a	0.79 (±0.54) a	0.00 (±0.00) a	1.57 (±1.07) a
18	9.13 (±1.73) b	3.93 (±2.68) ab	9.03 (±3.88) b	5.68 (±2.54) ab	2.06 (±1.41) a	4.71 (±2.06) ab	1.28 (±0.55) a	1.57 (±1.07) a
30	23.64 (±3.52) b	5.20 (±3.55) a	18.05 (±7.13) ab	12.94 (±4.46) ab	18.21 (±12.44) ab	7.16 (±2.56) a	4.02 (±2.04) a	1.96 (±0.99) a
42	30.02 (±4.37) d	8.24 (±3.99) ab	20.80 (±8.73) bcd	27.57 (±8.51) cd	20.37 (±10.69) bcd	10.89 (±3.46) abc	5.20 (±2.24) ab	1.96 (±0.99) a
54	37.36 (±4.82) c	12.46 (±4.25) ab	29.44 (±10.86) bc	34.54 (±7.98) c	20.76 (±10.96) abc	13.25 (±3.46) ab	5.20 (±2.24) a	1.96 (±0.99) a
66	43.73 (±5.03) bc	15.70 (±4.06) ab	38.84 (±14.27) bc	43.75 (±8.42) bc	46.77 (±23.96) c	16.78 (±4.27) abc	5.59 (±2.48) a	5.50 (±2.42) a
78	60.00 (±7.37) c	21.57 (±4.97) ab	41.19 (±15.33) bc	44.93 (±8.05) bc	50.01 (±23.20) bc	21.98 (±5.34) ab	5.59 (±2.48) a	7.46 (±3.30) a
90	72.65 (±9.97) d	28.74 (±7.15) abc	55.04 (±20.81) bcd	46.69 (±7.10) bcd	64.90 (±26.05) cd	24.72 (±6.42) ab	5.59 (±2.48) a	7.85 (±3.19) a
102	90.32 (±14.84) c	31.88 (±8.40) ab	60.63 (±22.88) bc	52.98 (±7.53) bc	68.82 (±25.41) bc	29.62 (±7.93) ab	5.59 (±2.48) a	9.03 (±3.28) a
114	95.41 (±16.26) d	42.96 (±13.81) abc	68.09 (±26.83) cd	59.65 (±6.50) bcd	70.39 (±25.23) cd	31.58 (±8.58) abc	5.59 (±2.48) a	14.92 (±7.20) ab

* Different letters in lines mark statistically significant differences (*p* ≤ 0.05).

**Table 6:** Selected physical and chemical properties of diatomite used in the experiment.

Dry Matter	Ash	pH H_2_O	EC *	BET Surface Area	Total Pore Volume	Zn	Pb	Cd	Cu	Ni
g·kg^−1^	g·kg^−1^		µS·cm^−1^	m^2^·g^−1^	cm^3^·g^−1^	mg·kg^−1^				
932.05 ± 4.01 ^1^	879.25 ± 2.11	5.76 ± 0.17	203.41 ± 6.23	31.30 ± 1.10	0.067 ± 0.002	36.82 ± 0.72	11.43 ± 0.04	0.16 ± 0.01	47.61 ± 1.93	17.52 ± 0.57

^1^ Each value represents the mean of three replicates ± SE. * EC—electrical conductivity.

## Data Availability

Data are contained within the article and Supplementary Materials.

## Supplementary Materials

The following supporting information can be downloaded at https://www.mdpi.com/article/10.3390/molecules30153300/s1: Table S1: Statistical analysis on the survival of *Aphis fabae* Scop. wingless females; Table S2: Statistical analysis on the survival of *Aphis fabae* Scop. nymphs; Table S3: Statistical analysis on the mass of leaves eaten by one female of *Leptinotarsa decemlineata* Say [g], no-choice experiment; Table S4: Statistical analysis on the mass of leaves eaten by one male of *Leptinotarsa decemlineata* Say [g], no-choice experiment; Table S5: Statistical analysis on survival of females of *Leptinotarsa decemlineata* Say, no-choice experiment; Table S6: Statistical analysis on survival of males of *Leptinotarsa decemlineata* Say, no-choice experiment; Table S7: Statistical analysis on body weight change of females and males of *Leptinotarsa decemlineata* Say [g], no-choice experiment; Table S8: Statistical analysis on the ADI value of *Leptinotarsa decemlineata* Say males and females, choice experiment; Table S9: Statistical analysis on the surface area of places eaten in leaves by one female of *Sitona lineatus* L., no-choice experiment; Table S10: Statistical analysis on the surface area of places eaten in leaves by one male of *Sitona lineatus* L., no-choice experiment; Table S11: Statistical analysis on the ADI value of *Sitona lineatus* L., choice experiment.

## References

[1] Pavela R. (2018). Essential oils from *Foeniculum vulgare* Miller as a safe environmental insecticide against the aphid *Myzus persicae* Sulzer. Environ. Sci. Pollut. Res..

[2] Benelli G., Pavela R., Zorzetto C., Sanchez-Mateo C.C., Santini G., Canale A., Maggi F. (2019). Insecticidal activity of the essential oil from *Schizogyne sericea* (Asteraceae) on four insect pests and two non-target species. Entomol. Gen..

[3] Parreira D.S., Alcantara-de la Cruz R., Zanuncio J.C., Lemes P.G., Rolim G.D., Barbosa L.R., Leite G.L.D., Serrao J.E. (2018). Essential oils cause detrimental effects on biological parameters of *Trichogramma galloi* immatures. J. Pest Sci..

[4] Kesraoui S., Andres M.F., Berrocal-Lobo M., Soudani S., Gonzalez-Coloma A. (2022). Direct and Indirect Effects of Essential Oils for Sustainable Crop Protection. Plants.

[5] Gondek K., Micek P., Baran A., Bajda T., Kowa L.J., Lis M., Wyrobisz-Papiewska A., Wojtysiak D., Smoroń K. (2023). Modified natural diatomite with various additives and its environmental potential. Materials.

[6] Gondek K., Baran A., Boguta P., Bołdak M. (2024). Use of diatomite-based composites for immobilization of toxic heavy metals in industrial wastes using post-flotation sediment as an example. Materials.

[7] Bakar H.E.G.M.M. (2010). Diatomite: Its chcracterization, modifications and applications. Asian J. Mater. Sci..

[8] Ikusika O.O., Mpendulo C.T., Zindove T.J., Okoh A.I. (2019). Fossil shell flour in livestock production: A review. Animals.

[9] Kaleta J., Papciak D., Puszkarewicz A. (2007). Clinoptylolite and diatomite respect of their usefulness for water conditioning and wastewater purification. Gospod. Surowcami Miner. Miner. Resour. Manag..

[10] Song X., Li C., Zhu Y., Yang Y., Chen M., Ma R., Ling X., Wu J. (2021). Application of diatomite for gallic acid removal from molasses wastewater. Sci. Total Environ..

[11] Aksakal E.L., Angin I., Oztas T. (2012). Effects of diatomite on soil physical properties. Catena.

[12] Ye X., Kang S., Wang H., Li H., Zhang Y., Wang G., Zhao H. (2015). Modified natural diatomite and its enhanced immobilization of lead, copper and cadmium in simulated contaminated soil. J. Hazard. Mater..

[13] Zeni V., Baliota G.V., Benelli G., Canale A., Athanassiou C.G. (2021). Diatomaceous Earth for Arthropod Pest Control: Back to the Future. Molecules.

[14] Vayias B.J., Athanassiou C.G., Koruni’c Z., Rozman V. (2009). Evaluation of natural diatomaceous earth deposits from south-eastern Europe for stored-grain protection: The effect of particle size. Pest Manag. Sci..

[15] Gad H., Atta A., Abdelgaleil S. (2022). Effectiveness of diatomaceous earth combined with chlorfluazuron and hexaflumuron in the control of *Callosobruchus maculatus* and *C. chinensis* on stored cowpea seeds. J. Stored Prod. Res..

[16] Gökçe M., Isikber A., Saglam Ö. (2021). Efficacy of Local Diatomaceous Earths Mixtures with Odorless Garlic Powder Against Confused Flour beetle, *Tribolium confusum* du Val. (Coleoptera: Tenebrionidae). Ksu Tarim Doga Derg..

[17] Gao Y., Yu S., Li J., Sun P., Xiong M., Lei C., Zhang Z., Huang Q. (2018). Bioactivity of diatomaceous earth against the subterranean termite *Reticulitermes chinensis* Snyder (Isoptera: Rhinotermitidae). Environ. Sci. Pollut. Res..

[18] Athanassiou C.G., Koruni’c Z. (2007). Evaluation of two new diatomaceous earth formulations, enhanced with abamectin and bitterbarkomycin, against four stored-grain beetle species. J. Stored Prod. Res..

[19] Singh B., Singh V. (2016). Laboratory and Field Studies Demonstrating the Insecticidal Potential of Diatomaceous Earth against Wheat Aphids in Rice-wheat Cropping System of Punjab (India). Cereal Res. Commun..

[20] Bounouira Y., Benyelles N.G., Senouci H., Benazzouz F.Z., Chaieb I. (2022). The insecticidal activity of a formulation of ammoides verticillata essential oil and diatomaceous earth on *Sitophilus zeamais*. Int. J. Trop. Insect Sci..

[21] Adarkwah C., Obeng-Ofori D., Hörmann V., Ulrichs C., Schöller M. (2017). Bioefficacy of enhanced diatomaceous earth and botanical powders on the mortality and progeny production of *Acanthoscelides obtectus* (Coleoptera: Chrysomelidae), *Sitophilus granarius* (Coleoptera: Dryophthoridae) and *Tribolium castaneum* (Coleoptera: Tenebrionidae) in stored grain cereals. Int. J. Trop. Insect Sci..

[22] Islam M., Hasan M., Lei C., Mucha-Pelzer T., Mewis I., Ulrichs C. (2010). Direct and admixture toxicity of diatomaceous earth and monoterpenoids against the storage pests *Callosobruchus maculatus* (F.) and *Sitophilus oryzae* (L.). J. Pest Sci..

[23] Krutzler M., Brader G., Madercic M., Riedle-Bauer M. (2022). Efficacy evaluation of alternative pest control products against Drosophila suzukii in Austrian elderberry orchards. J. Plant Dis. Prot..

[24] Deza-Borau G., Peschiutta M., Brito V., Usseglio V., Zunino M., Zygadlo J. (2020). Towards a development of novel bioinsecticides for organic control of Planococcus ficus in vineyards. Vitis.

[25] Kéïta S.M., Vincent C., Schmidt J.-P., Arnason J.T. (2001). Insecticidal effects of *Thuja occidentalis* (Cupressaceae) essential oil on *Callosobruchus maculatus* [Coleoptera: Bruchidae]. Can. J. Plant Sci..

[26] Hosseinzadeh J., Farazmand H., Karimpour Y. (2014). Insecticidal effect of *Thuja occidentalis* L. essential oilon adults of *Lasioderma serricorne* F. (Anobiidae) under laboratory conditions. Iran J. Med. Aromat. Pl..

[27] Abdelgaleil S.A.M., Badawy M.E.I., Shawir M.S., Mohamed M.I.E. (2015). Chemical Composition, Fumigant and Contact Toxicities of Essential Oils Isolated from Egyptian Plants against the Stored Grain Insects *Sitophilus oryzae* L. and *Tribolium castaneum* (Herbst). Egypt. J. Biol. Pest Control.

[28] Brari J., Thakur D.R. (2018). Larvicidal effects of eight essential oils against *Plodia interpunctella* and *Tribolium castaneum*, serious pests of stored products worldwide. J. Entomol. Zool. Stud..

[29] Martynov V.O., Titov O.G., Kolombar T.M., Brygadyrenko V.V. (2019). Influence of essential oils of plants on the migration activity of *Tribolium confusum* (Coleoptera, Tenebrionidae). Biosyst. Divers..

[30] Pavela R. (2008). Insecticidal properties of several essential oils on the house fly (*Musca domestica* L.). Phytother. Res..

[31] Benelli G., Flamini G., Canale A., Cioni P.L., Conti B. (2012). Toxicity of some essential oil formulations against the Mediterranean fruit fly *Ceratitis capitata* (Wiedemann) (Diptera Tephritidae). Crop Protect..

[32] Gospodarek J., Krajewska A., Pasmionka I., Bruzdzinska J., Tamiru G. (2024). Potential of *Thuja occidentalis* L. Essential Oil and Water Extracts against Field Crop Pests. Molecules.

[33] Lis A., Liśkiewicz R., Krajewska A. (2016). Comparison of chemical composition of essentials oils from different parts of *Thuja occidentalis* L. ‘Brabant’ and *T. occidentalis* L. ‘Smaragd’. Herba Pol..

[34] Caruntu S., Ciceu A., Olah N.K., Don I., Hermenean A., Cotoraci C. (2020). *Thuja occidentalis* L. (Cupressaceae): Ethnobotany, Phytochemistry and Biological Activity. Molecules.

[35] Bai L., Wang W., Hua J., Guo Z., Luo S. (2020). Defensive functions of volatile organic compounds and essential oils from northern white-cedar in China. BMC Plant Biol..

[36] Fitsev I.M., Nikitin E.N., Rakhmaeva A.M., Terenzhev D.A., Sakhno T.M., Nasybullina Z.R. (2022). Chemical Composition of *Cupressus sempervirens* L. and *Thuja occidentalis* L. Essential Oils and Their Activity against Phytopathogenic Fungi. Uchenye Zap. Kazan. Univ.-Seriya Estestv. Nauk..

[37] Zhu Y., Stahl A., Rostas M., Will T. (2023). Temporal and species-specific resistance of sugar beet to green peach aphid and black bean aphid: Mechanisms and implications for breeding. Pest Manag. Sci..

[38] Shannag H.K., Ababneh J.A. (2007). Influence of black bean aphid, *Aphis fabae* Scopoli on growth rates of faba bean. World J. Agric. Sci..

[39] Hanavan R.P., Bosque-Pérez N.A. (2012). Effects of tillage practices on pea leaf weevil (*Sitona lineatus* L., Coleoptera: Curculionidae) biology and crop damage: A farm-scale study in the US Pacific Northwest. Bull. Entomol. Res..

[40] Lohaus K., Vidal S. (2010). Abundance of *Sitona lineatus* L. (Col., Curculionidae) in peas (Pisum sutivum L.): Effects on yield parameters and nitrogen balance. Crop Protect..

[41] EPPO (2025). *Leptinotarsa decemlineata*. EPPO Datasheets on Pests Recommended for Regulation. https://gd.eppo.int.

[42] Gospodarek J., Krajewska A., Pasmionka I.B. (2023). Contact and Gastric Effect of Peppermint Oil on Selected Pests and Aphid Predator *Harmonia axyridis* Pallas. Molecules.

[43] Gospodarek J., Endalamew A., Worsdale M., Pasmionka I. (2022). Effects of *Artemisia dracunculus* L. Water Extracts on Selected Pests and Aphid Predator *Coccinella septempunctata* L. Agronomy.

[44] Valizadeh H., Abbasipour H., Farazmand H., Askarianzadeh A. (2013). Evaluation of Kaolin Application on Oviposition Control of the Vine Cicada, Psalmocharias alhageos in Vineyards Homoptera: Cicadidae. Entomol. Gen..

[45] Hernández-Suárez E., Arjona-López J., Rizza R., Perera S., Siverio F., Hervalejo A., Arenas-Arenas F. (2023). Comparative efficacy of seven biorational insecticides to manage African citrus psyllid (*Trioza erytreae*) in European organic citriculture. Biol. Agric. Hortic..

[46] Paponja I., Rozman V., Liska A. (2020). Natural Formulation Based on Diatomaceous Earth and Botanicals against Stored Product Insects. Insects.

[47] Bachrouch O., Nefzi H., Belloumi S., Horchani-Naifer K., Eljazi J., Hamdi S., Msaada K., Labidi J., Abderrabba M., Ben Jemaa J. (2023). Insecticidal effects of two Tunisian diatomaceous earth loaded with *Thymus capitatus* (L.) Hoffmans and Links as an ecofriendly approach for stored coleopteran pest control. Int. J. Environ. Health Res..

[48] Atay T., Alkan M., Ertürk S., Toprak U. (2023). Individual and combined effects of α-Pinene and a native diatomaceous earth product on control of stored product beetle pests. J. Asia-Pac. Entomol.

[49] Batistic L., Bohinc T., Horvat A., Kosir I., Trdan S. (2023). Laboratory Investigation of Five Inert Dusts of Local Origin as Insecticides against the Colorado Potato Beetle (*Leptinotarsa decemlineata* [Say]). Agronomy.

[50] Prasantha B., Reichmuth C., Adler C. (2019). Lethality and kinetic of diatomaceous earth uptake by the bean weevil (*Acanthoscelides obtectus* [Say] Coleoptera: Bruchinae): Influence of short-term exposure period. J. Stored Prod. Res..

[51] de Paula M., Flinn P., Subramanyam B., Lazzari S. (2002). Effects of age and sex on mortality of *Tribolium castaneum* (Herbst) (Coleoptera: Tenebrionidae) exposed to INSECTO^®^-treated wheat. J. Kans. Entomol. Soc..

[52] Lazarevic J., Jevremovic S., Kostic I., Vuleta A., Jovanovic S., Kostic M., Jovanovic D. (2022). Assessment of Sex-Specific Toxicity and Physiological Responses to Thymol in a Common Bean Pest *Acanthoscelides obtectus* Say. Front. Physiol..

[53] Pavela R., Maggi F., Mazzara E., Torresi J., Cianfaglione K., Benelli G., Canale A. (2021). Prolonged sublethal effects of essential oils from non-wood parts of nine conifers on key insect pests and vectors. Ind. Crops Prod..

[54] Kwiecien N., Gospodarek J., Boliglowa E. (2020). The Effects of Water Extracts from Tansy on Pea Leaf Weevil and Black Bean Aphid. J. Ecol. Eng..

[55] Finney D. (1971). Probit Analysis.

