# Supplementary material for: Effect of Thuja occidentalis L. Essential Oil Combined with Diatomite Against Selected Pests
Source: Molecules. 2025 Aug 6;30(15):3300. doi: 10.3390/molecules30153300 (PMC12348420; doi:10.3390/molecules30153300)
Supplement: Supplementary file 1 [file molecules-30-03300-s001.zip › molecules-3744872-supplementary (1).pdf]

Table S1. Statistical analysis on the survival of *Aphis fabae* Scop. wingless females.

	df	Sum of Squares	Mean Square	F	p
			6 h		
EO	3	89553.5	29851.2	365.773	0.000000
DE	2	782.3	391.2	4.793	0.011718
EO*DE	6	2467.0	411.2	5.038	0.000306
			18 h		
EO	3	87673.5	29224.5	338.726	0.000000
DE	2	702.3	351.2	4.070	0.021999
EO*DE	6	2563.0	427.2	4.951	0.000356
			30 h		
EO	3	92157.5	30719.2	356.050	0.000000
DE	2	450.3	225.2	2.610	0.081884
EO*DE	6	1759.0	293.2	3.398	0.005950
			42 h		
EO	3	86709.5	28903.2	218.504	0.000000
DE	2	132.3	66.2	0.500	0.608910
EO*DE	6	2317.0	386.2	2.919	0.014501
			54 h		
EO	3	90456.2	30152.1	425.342	0.000000
DE	2	554.8	277.4	3.913	0.025272
EO*DE	6	1812.3	302.1	4.261	0.001222
			66 h		
EO	3	74381.5	24793.8	239.062	0.000000
DE	2	2021.7	1010.9	9.747	0.000216
EO*DE	6	3209.2	534.9	5.157	0.000248
			78 h		
EO	3	59117.6	19705.9	126.445	0.000000
DE	2	2028.3	1014.2	6.508	0.002768
EO*DE	6	4486.5	747.8	4.798	0.000467
			90 h		
EO	3	45159.1	15053.0	100.1336	0.000000
DE	2	1730.5	865.2	5.7557	0.005168
EO*DE	6	5271.0	878.5	5.8438	0.000076
			102 h		
EO	3	34720.43	11573.48	74.4359	0.000000
DE	2	1743.76	871.88	5.6076	0.005853
EO*DE	6	5807.06	967.84	6.2248	0.000040
			114 h		
EO	3	23537.46	7845.82	51.0526	0.000000
DE	2	1910.80	955.40	6.2168	0.003518
EO*DE	6	3920.67	653.45	4.2520	0.001242

Table S2. Statistical analysis on the survival of *Aphis fabae* Scop. nymphs.

	df	Sum of Squares	Mean Square	F	p
			6 h		
EO	3	134340.7	44780.2	27204.0	0.000000
DE	2	4.9	2.5	1.5	0.231377
EO*DE	6	14.8	2.5	1.5	0.193685
			18 h		
EO	3	117084.2	39028.1	417.411	0.000000
DE	2	1446.6	723.3	7.736	0.001026
EO*DE	6	4534.3	755.7	8.083	0.000002
			30 h		

EO	3	110072.4	36690.8	264.377	0.000000
DE	2	1069.0	534.5	3.851	0.026694
EO*DE	6	4060.8	676.8	4.877	0.000406
			42 h		
EO	3	105797.7	35265.9	182.741	0.000000
DE	2	671.5	335.7	1.740	0.184300
EO*DE	6	3354.0	559.0	2.897	0.015129
			54 h		
EO	3	105185.8	35061.9	180.872	0.000000
DE	2	233.4	116.7	0.602	0.550941
EO*DE	6	1917.6	319.6	1.649	0.149567
			66 h		
EO	3	94071.4	31357.1	131.8145	0.000000
DE	2	16.6	8.3	0.0350	0.965643
EO*DE	6	1221.3	203.6	0.8557	0.532638
			78 h		
EO	3	86540.8	28846.9	137.6780	0.000000
DE	2	374.1	187.0	0.8927	0.414913
EO*DE	6	2953.0	492.2	2.3490	0.041948
			90 h		
EO	3	76492.8	25497.6	133.4452	0.000000
DE	2	888.4	444.2	2.3248	0.106556
EO*DE	6	4868.7	811.4	4.2468	0.001254
			102 h		
EO	3	65617.09	21872.36	100.7717	0.000000
DE	2	895.47	447.73	2.0628	0.136014
EO*DE	6	5126.72	854.45	3.9367	0.002205
			114 h		
EO	3	53625.15	17875.05	112.3191	0.000000
DE	2	1260.81	630.41	3.9612	0.024219
EO*DE	6	4293.31	715.55	4.4962	0.000800

Table S3. Statistical analysis on the mass of leaves eaten by one female of *Leptinotarsa decemlineata* Say [g], no-choice experiment.

	df	Sum of Squares	Mean Square	F	p
			24 h		
EO	3	0.072184	0.024061	2.28904	0.097891
DE	1	0.205189	0.205189	19.52031	0.000113
EO*DE	3	0.189404	0.063135	6.00622	0.002384
			48 h		
EO	3	0.248831	0.082944	5.00974	0.006011
DE	1	0.171735	0.171735	10.37266	0.002999
EO*DE	3	0.210605	0.070202	4.24013	0.012720
			72 h		
EO	3	0.492283	0.164094	2.49442	0.078274
DE	1	0.010773	0.010773	0.16376	0.688494
EO*DE	3	0.301546	0.100515	1.52795	0.226797
			96 h		
EO	3	0.76298	0.25433	3.2760	0.034024
DE	1	0.00352	0.00352	0.0453	0.832865
EO*DE	3	0.56518	0.18839	2.4267	0.084248

Table S4. Statistical analysis on the mass of leaves eaten by one male of *Leptinotarsa decemlineata* Say [g], no-choice experiment.

	df	Sum of Squares	Mean Square	F	p
			24 h		
EO	3	0.119951	0.039984	5.1129	0.004752
DE	1	0.922342	0.922342	117.9437	0.000000
EO*DE	3	0.129460	0.043153	5.5182	0.003189
			48 h		
EO	3	0.180533	0.060178	3.66442	0.021081
DE	1	1.099571	1.099571	66.95644	0.000000
EO*DE	3	0.164683	0.054894	3.34269	0.029755
			72 h		
EO	3	0.748602	0.249534	9.4792	0.000094
DE	1	0.079941	0.079941	3.0367	0.089937
EO*DE	3	0.451605	0.150535	5.7184	0.002626
			96 h		
EO	3	1.35460	0.45153	8.6011	0.000195
DE	1	0.24615	0.24615	4.6887	0.037065
EO*DE	3	0.40810	0.13603	2.5912	0.067761

Table S5. Statistical analysis on survival of females of *Leptinotarsa decemlineata* Say, no-choice experiment.

	df	Sum of Squares	Mean Square	F	p
			24 h		
EO	3	0.120000	0.040000	1.000000	0.402797
DE	1	0.000000	0.000000	0.000000	1.000000
EO*DE	3	0.240000	0.080000	2.000000	0.129442
			48 h		
EO	3	0.240000	0.080000	0.57143	0.637098
DE	1	0.120000	0.120000	0.85714	0.360092
EO*DE	3	0.600000	0.200000	1.42857	0.248666
			72 h		
EO	3	0.330000	0.110000	0.61111	0.611751
DE	1	0.270000	0.270000	1.50000	0.227835
EO*DE	3	0.330000	0.110000	0.61111	0.611751
			96 h		
EO	3	1.080000	0.360000	2.00000	0.129442
DE	1	0.120000	0.120000	0.66667	0.419052
EO*DE	3	1.080000	0.360000	2.00000	0.129442

Table S6. Statistical analysis on survival of males of *Leptinotarsa decemlineata* Say, no-choice experiment.

	df	Sum of Squares	Mean Square	F	p
			24 h		
EO	3	0.120000	0.040000	1.000000	0.402797
DE	1	0.000000	0.000000	0.000000	1.000000
EO*DE	3	0.240000	0.080000	2.000000	0.129442
			48 h		
EO	3	0.330000	0.110000	2.200000	0.103018
DE	1	0.030000	0.030000	0.600000	0.443133
EO*DE	3	0.570000	0.190000	3.800000	0.017319
			72 h		
EO	3	0.720000	0.240000	3.428571	0.025971

DE	1	0.000000	0.000000	0.000000	1.000000
EO*DE	3	0.240000	0.080000	1.142857	0.343497
			96 h		
EO	3	2.490000	0.830000	6.91667	0.000736
DE	1	0.030000	0.030000	0.25000	0.619815
EO*DE	3	0.090000	0.030000	0.25000	0.860855

Table S7. Statistical analysis on body weight change of females and males of *Leptinotarsa decemlineata* Say [g], no-choice experiment.

	df	Sum of Squares	Mean Square	F	p
			females		
EO	3	0.000729	0.000243	0.4926	0.689410
DE	1	0.001728	0.001728	3.5032	0.068578
EO*DE	3	0.002498	0.000833	1.6880	0.184946
			males		
EO	3	0.000134	0.000045	0.16646	0.918362
DE	1	0.000094	0.000094	0.35050	0.557165
EO*DE	3	0.002365	0.000788	2.93659	0.044810

Table S8. Statistical analysis on the ADI value of *Leptinotarsa decemlineata* Say males and females, choice experiment.

	df	Sum of Squares	Mean Square	F	p
			24 h		
Treatment (T)	4	61810.4	15452.6	17.7646	0.000000
Sex	1	4467.0	4467.0	5.1354	0.031999
T*Sex	4	17126.8	4281.7	4.9223	0.004333
			48 h		
Treatment (T)	4	57061.7	14265.4	12.6344	0.000008
Sex	1	3983.6	3983.6	3.5281	0.071595
T*Sex	4	14505.9	3626.5	3.2118	0.028653
			72 h		
Treatment (T)	4	42130.0	10532.5	7.53353	0.000360
Sex	1	3555.8	3555.8	2.54331	0.122848
T*Sex	4	14874.8	3718.7	2.65986	0.055245
			96 h		
Treatment (T)	4	19943.63	4985.91	3.11822	0.031979
Sex	1	1588.75	1588.75	0.99362	0.328048
T*Sex	4	19694.55	4923.64	3.07928	0.033480

Table S9. Statistical analysis on the surface area of places eaten in leaves by one female of *Sitona lineatus* L., no-choice experiment.

	df	Sum of Squares	Mean Square	F	p
			6 h		
EO	3	0.346627	0.115542	2.142857	0.109956
DE	1	0.115542	0.115542	2.142857	0.151050
EO*DE	3	0.346627	0.115542	2.142857	0.109956
			18 h		
EO	3	5109.91	1703.30	1.50231	0.228634
DE	1	2785.12	2785.12	2.45647	0.124919
EO*DE	3	3059.90	1019.97	0.89961	0.449860
			30 h		
EO	3	10660.2	3553.4	2.76650	0.054226

DE	1	1564.7	1564.7	1.21823	0.276304
EO*DE	3	926.0	308.7	0.24031	0.867703
			42 h		
EO	3	45175.3	15058.4	6.58699	0.001007
DE	1	309.2	309.2	0.13527	0.714970
EO*DE	3	3681.6	1227.2	0.53681	0.659773
			54 h		
EO	3	54489.9	18163.3	7.4065	0.000465
DE	1	1249.0	1249.0	0.5093	0.479573
EO*DE	3	1597.8	532.6	0.2172	0.883905
			66 h		
EO	3	56203.0	18734.3	8.3600	0.000196
DE	1	2260.9	2260.9	1.0089	0.321203
EO*DE	3	4451.0	1483.7	0.6621	0.580258
			78 h		
EO	3	52464.2	17488.1	7.6853	0.000360
DE	1	653.3	653.3	0.2871	0.595055
EO*DE	3	2240.5	746.8	0.3282	0.804956
			90 h		
EO	3	69554.7	23184.9	10.6349	0.000028
DE	1	123.0	123.0	0.0564	0.813475
EO*DE	3	3661.3	1220.4	0.5598	0.644650
			102 h		
EO	3	78393.5	26131.2	11.2218	0.000018
DE	1	348.8	348.8	0.1498	0.700771
EO*DE	3	4698.8	1566.3	0.6726	0.573885
			114 h		
EO	3	80582.7	26860.9	10.8905	0.000023
DE	1	316.1	316.1	0.1282	0.722215
EO*DE	3	9686.0	3228.7	1.3090	0.284811

Table S10. Statistical analysis on the surface area of places eaten in leaves by one male of *Sitona lineatus* L., no-choice experiment.

	df	Sum of Squares	Mean Square	F	p
			6 h		
EO	3	54.4998	18.16660	1.198736	0.322593
DE	1	20.2849	20.28488	1.338511	0.254159
EO*DE	3	112.1554	37.38512	2.466883	0.076056
			18 h		
EO	3	274.149	91.383	3.09851	0.037407
DE	1	23.567	23.567	0.79909	0.376712
EO*DE	3	112.427	37.476	1.27069	0.297441
			30 h		
EO	3	1179.28	393.095	1.99983	0.129467
DE	1	1007.62	1007.623	5.12617	0.029061
EO*DE	3	469.87	156.625	0.79681	0.502951
			42 h		
EO	3	2768.52	922.84	3.89464	0.015635
DE	1	576.43	576.43	2.43269	0.126707
EO*DE	3	1285.28	428.43	1.80808	0.161211
			54 h		
EO	3	5337.48	1779.16	6.57127	0.001023
DE	1	699.92	699.92	2.58513	0.115737
EO*DE	3	1438.96	479.65	1.77158	0.168085
			66 h		

EO	3	8340.79	2780.26	4.02480	0.013592
DE	1	2123.40	2123.40	3.07391	0.087219
EO*DE	3	3005.23	1001.74	1.45016	0.242631
			78 h		
EO	3	10366.32	3455.44	4.78624	0.006083
DE	1	2777.07	2777.07	3.84661	0.056834
EO*DE	3	4062.23	1354.08	1.87557	0.149234
			90 h		
EO	3	16215.41	5405.14	5.26042	0.003736
DE	1	6098.77	6098.77	5.93548	0.019386
EO*DE	3	4752.94	1584.31	1.54190	0.218541
			102 h		
EO	3	21952.72	7317.57	6.11162	0.001597
DE	1	7781.02	7781.02	6.49870	0.014734
EO*DE	3	7286.72	2428.91	2.02862	0.125277
			114 h		
EO	3	25601.4	8533.8	5.73176	0.002325
DE	1	6125.0	6125.0	4.11386	0.049228
EO*DE	3	7120.5	2373.5	1.59417	0.205882

Table S11. Statistical analysis on the ADI value of *Sitona lineatus* L., choice experiment.

	df	Sum of Squares	Mean Square	F	p
			30 h		
Treatment (T)	4	11042.2	2760.5	1.19124	0.345072
Sex	1	551.8	551.8	0.23810	0.630887
T*Sex	4	6414.3	1603.6	0.69199	0.606145
			42 h		
Treatment (T)	4	13391.9	3348.0	1.34648	0.287574
Sex	1	736.3	736.3	0.29611	0.592346
T*Sex	4	9003.6	2250.9	0.90527	0.479664
			54 h		
Treatment (T)	4	12053.9	3013.5	1.19687	0.342809
Sex	1	540.0	540.0	0.21447	0.648284
T*Sex	4	8803.3	2200.8	0.87411	0.496756
			66 h		
Treatment (T)	4	8516.0	2129.0	0.91590	0.473941
Sex	1	1150.3	1150.3	0.49487	0.489871
T*Sex	4	8720.3	2180.1	0.93787	0.462300
			78 h		
Treatment (T)	4	7490.1	1872.5	0.81125	0.532710
Sex	1	1762.6	1762.6	0.76363	0.392567
T*Sex	4	8555.2	2138.8	0.92662	0.468232
			90 h		
Treatment (T)	4	7457.6	1864.4	0.80625	0.535651
Sex	1	1593.4	1593.4	0.68905	0.416285
T*Sex	4	8491.1	2122.8	0.91799	0.472822
			102 h		
Treatment (T)	4	6669.0	1667.2	0.72722	0.583770
Sex	1	1898.9	1898.9	0.82828	0.373610
T*Sex	4	8706.9	2176.7	0.94945	0.456261
			114 h		
Treatment (T)	4	5574.1	1393.5	0.61477	0.656972
Sex	1	2540.3	2540.3	1.12071	0.302386
T*Sex	4	8192.8	2048.2	0.90359	0.480572
